# Incidence and real-world burden of brain metastases from solid tumors and hematologic malignancies in Ontario: a population-based study

**DOI:** 10.1093/noajnl/vdaa178

**Published:** 2020-12-22

**Authors:** Steven Habbous, Katharina Forster, Gail Darling, Katarzyna Jerzak, Claire M B Holloway, Arjun Sahgal, Sunit Das

**Affiliations:** 1 Ontario Health (Cancer Care Ontario), Toronto, Ontario, Canada; 2 Faculty of Medicine, University of Toronto, Toronto, Ontario, Canada; 3 Division of Thoracic Surgery, Toronto General Hospital, Toronto, Ontario, Canada; 4 Division of Medical Oncology, Sunnybrook Health Sciences Centre, Toronto, Ontario, Canada; 5 Department of Surgery, Sunnybrook Health Sciences Centre, Toronto, Ontario, Canada; 6 Department of Radiation Oncology, Sunnybrook Health Sciences Centre, Toronto, Ontario, Canada; 7 Division of Neurosurgery, St. Michael’s Hospital, Toronto, Ontario, Canada

**Keywords:** brain imaging, brain metastases, incidence, intracranial metastatic disease

## Abstract

**Background:**

Although intracranial metastatic disease (IMD) is a frequent complication of cancer, most cancer registries do not capture these cases. Consequently, a data-gap exists, which thwarts system-level quality improvement efforts. The purpose of this investigation was to determine the real-world burden of IMD.

**Methods:**

Patients diagnosed with a non-CNS cancer between 2010 and 2018 were identified from the Ontario Cancer Registry. IMD was identified by scanning hospital administrative databases for cranial irradiation or coding for a secondary brain malignancy (ICD-10 code C793).

**Results:**

25,478 of 601,678 (4.2%) patients with a diagnosis of primary cancer were found to have IMD. The median time from primary cancer diagnosis to IMD was 5.2 (0.7, 15.4) months and varied across disease sites, for example, 2.1 months for lung, 7.3 months for kidney, and 22.8 months for breast. Median survival following diagnosis with IMD was 3.7 months. Lung cancer accounted for 60% of all brain metastases, followed by breast cancer (11%) and melanoma (6%). More advanced stage at diagnosis and younger age were associated with a higher likelihood of developing IMD (*P* < .0001). IMD was also associated with triple-negative breast cancers and ductal histology (*P* < .001), and with small-cell histology in patients with lung cancer (*P* < .0001). The annual incidence of IMD was 3,520, translating to 24.2 per 100,000 persons.

**Conclusion:**

IMD represents a significant burden in patients with systemic cancers and is a significant cause of cancer mortality. Our findings support measures to actively capture incidents of brain metastasis in cancer registries.

Key Points

- Cancer registries rarely capture and report on the incidence of brain metastasis, leaving a data-gap that thwarts system-level quality improvement efforts.
- In this study, we provide population-level data on the incidence of brain metastases, risk factors, and outcomes following its discovery.
- Building on these methods, health systems can allocate resources for prevention and surveillance strategies to mitigate the complications associated with intracranial metastatic disease.

**Importance of the Study**

Because cancer registries do not capture brain metastasis, the population-level burden of intracranial metastatic disease is difficult to quantify. Our analysis provides disease-site specific estimates of the risk of brain metastasis in patients diagnosed in Ontario with a primary cancer diagnosis with up to 11 years of follow-up. We estimate that brain metastasis occurs at a rate of 24.2 per 100,000 persons per year – higher than all other primary brain cancers combined. The findings of our study will be critical to efforts to improve health service delivery in Ontario for cancer patients, but are likely informative of the burden of brain metastasis beyond our geographic jurisdiction.

Intracranial metastatic disease (IMD) is a frequent complication of cancer. With incidence estimates at least as large as all primary brain tumors combined, the burden on patients and healthcare systems is significant.^1,2^ Unfortunately, most cancer registries do not capture cases of brain metastases (BM).^3^ The lack of data impairs efforts to track incidence, monitor performance, and plan health systems. Most estimates come from historical studies prior to the MRI era, and many are based on autopsy reports that significantly underrepresent the actual number of cases.^2,4^ More recent studies provide estimates around 10–20 per 100,000.^5^ As populations continue to age and cancer survivorship improves, the incidence of IMD is expected to rise.^2,6^ Estimating the incidence and timing of BM is critical to inform system-level changes.^7–9^

Lung, breast, melanoma, kidney, and colorectal cancers have historically accounted for the majority of all BM.^10,11^ Although this finding is attributable in large part to the prevalence of these cancers, certain subgroups of primary cancers do have a higher biological propensity towards metastatic spread to the brain. Metastasis rates may also vary according to tumor size, nodal involvement, histology, and genetic/epigenetic factors.^12–14^ Identifying risk factors for BM are important to inform treatment strategies for the primary cancer to prevent IMD, and planning schedules for follow-up screening for earlier detection to maximize opportunities to achieve intracranial disease control and prolonged survival.^7,8,15–17^

In this study, we screened cases of non-CNS cancer from the Ontario Cancer Registry between 2010 and 2018 to determine the population-level incidence of IMD in Ontario, Canada.

## Methods

### Cohort Ascertainment

We identified adults (age 18+) with an incident solid or hematologic non-brain cancer from the Ontario Cancer Registry (OCR) diagnosed between January 1, 2010 and December 31, 2018. Cases of primary CNS tumors (ICD-O-3 topography codes C700-C729) were excluded. Only Ontario residents (eg, patients with a valid health card number and an Ontario postal code at the time of diagnosis) were included in study.

### Indicators for Brain Metastasis

To identify brain resection, we searched the Discharge Abstract Database (DAD) and the National Ambulatory Care Reporting System (NACRS) databases for hospital-based procedures (Supplementary Table S1 for surgical codes). To identify brain radiation, we searched DAD, NACRS, and the Activity Level Reporting (ALR) database for radiation to the head. All regional cancer centers provided data to Ontario Health through the ALR system on provision of radiation throughout the study period. Lastly, we searched DAD and NACRS for any procedure performed with the ICD-10 diagnostic code C793 (“Secondary malignant neoplasm of brain and cerebral meninges”).

### Outcomes

We estimated the time from diagnosis of the primary cancer (derived from the OCR) until IMD. We also estimated the overall survival of cancer patients starting from the time of their IMD diagnosis until the date of last follow-up or death. The fact/date of death was obtained from the OCR and the Registered Persons Database. Patients were followed until December 31, 2019 for IMD or death.

### Other Covariates

#### Sociodemographics

Patient age and sex were obtained from the OCR. Patient sociodemographic factors were obtained from the 2016 Canadian Census using the patients’ postal code at the time of diagnosis. Using the Postal Code Conversion File, neighborhood-level covariates were derived, including neighborhood after-tax income quintile and rurality.

#### Tumor characteristics

Topography and histology were obtained from the OCR and initially classified as per the SEER recode. SEER recode groups belonging to the head and neck (20010–20100, 22010, 22020) and miscellaneous (37000) were recoded based on the ICD-O-3 topography codes (Supplementary Table S2). Cancer stage was obtained from the Collaborative Staging database at Ontario Health, which is derived from the best available evidence of stage for lung, breast, colorectal, and prostate cancers. The Collaborative Staging database also includes site-specific factors for select disease sites. For prostate cancer, prostate-specific antigen (PSA) and Gleason score were obtained. For breast cancers, patients were classified according to the human epidermal growth factor receptor2 (HER2) and hormone (estrogen and progesterone) receptor (HR) status: (1) triple-negative (HR- and HER2-); (2) HR- and HER2+; (3) HR+ (either estrogen or progesterone) and HER2-; and (4) HR+ and HER2+.

#### Clinical characteristics

To estimate comorbidity, we used the Charlson Comorbidity Index, searching 3 years before diagnosis and excluding cancer (DAD and NACRS). Patients with no hospital visit (missing comorbidity) were considered to have a distinct comorbidity category (Supplementary Figure S1).

### Statistical Methods, Software, and Privacy

Mean (SD), median (interquartile range [IQR]: 25th, 75th percentile), and *N* (percent) were presented where appropriate. Time-to-event analyses were presented using Kaplan–Meier plots and Cox proportional hazards regression, reporting hazard ratios (HR) with 95% confidence intervals (CI). We also used logistic regression to estimate the likelihood of developing a brain metastasis, reporting odds ratios (OR) with 95% CI. We used Statistical Analysis Software v9.4 (SAS) for all analyses. To comply with privacy requirements, data were suppressed if the number of patients was <6. Ethics approval was not required.

## Results

**Figure 1.**
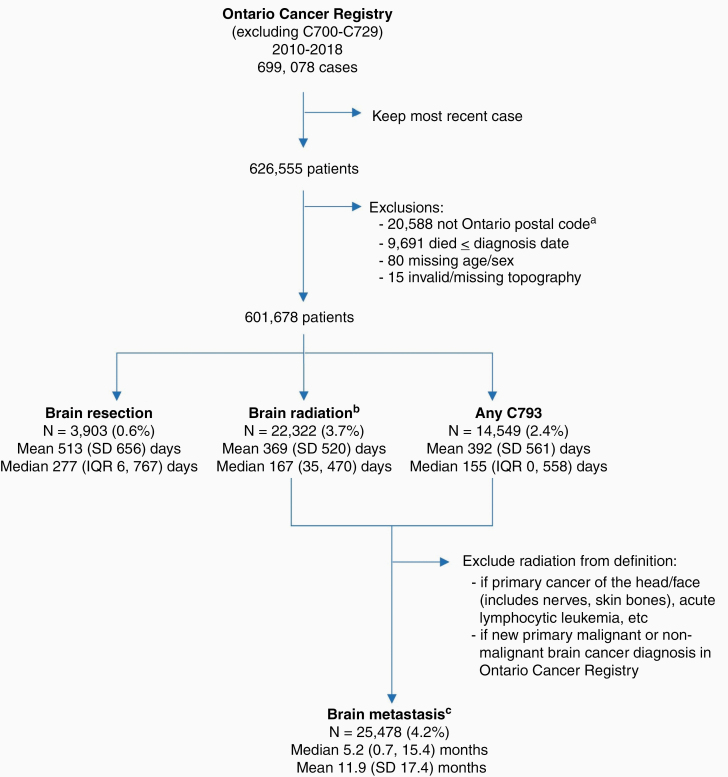
Cohort creation. Cohort creation from the Ontario Cancer Registry (OCR), excluding primary cancers of the brain and CNS (ICD Oncology version 3 codes C70-C72). Means and medians are the time from primary cancer diagnosis until brain metastasis was identified. ^a^Postal code was derived from the OCR, which corresponds to the patients’ postal code at the time of diagnosis. ^b^709 identified from CIHI databases only; 4,379 identified from the Activity Level Reporting database only. ICD-10—International Classification of Diseases, 10th revision; C793—ICD-10 diagnostic code “Secondary malignant neoplasm of brain and cerebral meninges”; IQR—25th, 75th percentile.

After applying exclusions, we observed a total 601,678 new incident solid and hematologic cancer diagnoses in Ontario between 2010 and 2018 (Figure 1). The most common cancers arose in the breast (*N* = 87,175; 14%), lung and bronchus (*n* = 77,211; 13%), and prostate (*n* = 71,311; 12%) (Table 1). The mean age was 67 (SD 14.3) years at the time of primary tumor diagnosis. The cohort was evenly split by sex (males, *n* = 304,446, 50.6%; females, *n* = 297,232; 49.4%). The majority of patients resided in an urban area (*n* = 520,031; 86%).

**Table 1.** Brain Metastasis and Timing by Disease Site

Disease Site (SEER recode)	*N* (%) of All Cancers 2010–2018	*N* (%) with Brain Metastases	Median (IQR) Time Until Metastasis (months)	1-year All- cause Mortality	Median (IQR) Time to Death Since Metastasis (months)^d^
Head and neck					
Lip (20010)	832 (0.2%)	<6	–	7.9%	–
Salivary glands^a^	1,765 (0.3%)	65 (3.7%)	13.6 (7.8, 27.3)	18.1%	4.9 (1.5, 11.3)
Nasopharynx (20060)^a^	898 (0.1%)	42 (4.7%)	7.5 (2.3, 21.3)	16.3%	6.0 (1.4, 20.2)
Nose and nasal sinus^a,b^	810 (0.1%)	28 (3.5%)	13.2 (8.1, 24.5)	22.2%	3.3 (0.8, 7.3)
Oropharynx (20080)^a^	4,535 (0.8%)	77 (1.7%)	15.7 (6.2, 31.8)	18.4%	2.6 (1.0, 6.3)
Hypopharynx (20090)^a^	613 (0.1%)	14 (2.3%)	14.7 (3.7, 29.4)	43.7%	2.7 (1.8, 8.4)
Oral cavity^a^	5,825 (0.9%)	93 (1.6%)	7.6 (2.8, 20.8)	22.6%	3.3 (1.5, 14.3)
Larynx^a^	3,055 (0.5%)	35 (1.2%)	11.2 (2.7, 20.5)	18.9%	2.1 (1.0, 6.6)
Digestive system					
Esophagus (21010)	6,415 (1.1%)	333 (5.2%)	5.9 (1.1, 13.7)	57.9%	2.3 (1.1, 5.6)
Stomach (21020)	11,878 (2%)	254 (2.1%)	8.8 (1.3, 19.0)	48.4%	2.2 (1.0, 5.6)
Small intestine (21030)	3,471 (0.6%)	32 (1.0%)	4.0 (1.0, 23.8)	28.6%	2.7 (1.1, 11.3)
Cecum (21041)	10,302 (1.7%)	129 (1.3%)	16.9 (3.9, 33.7)	22.4%	2.3 (1.3, 6.0)
Appendix (21042)	1,778 (0.3%)	<6	–	7%	–
Ascending colon (21043)	8,378 (1.4%)	108 (1.3%)	16.2 (5.3, 31.9)	19.3%	2.9 (1.2, 7.0)
Hepatic flexure (21044)	1,941 (0.3%)	15 (0.8%)	11.7 (4.0, 37.2)	26.5%	1.7 (1.4, 2.5)
Transverse colon (21045)	3,814 (0.6%)	31 (0.8%)	10.8 (4.4, 32.9)	24.1%	2.7 (0.8, 9.6)
Splenic flexure (21046)	1,345 (0.2%)	14 (1.0%)	22.7 (8.2, 38.6)	22.5%	2.7 (2.0, 4.6)
Descending colon (21047)	2,479 (0.4%)	31 (1.3%)	21.9 (3.2, 39.1)	18.6%	1.9 (1.2, 3.9)
Sigmoid colon (21048)	11,415 (1.9%)	123 (1.1%)	23.7 (6.4, 40.4)	17.5%	2.0 (1.2, 5.5)
Large intestine, NOS (21049)	2,093 (0.3%)	78 (3.7%)	1.0 (0.0, 14.8)	65.6%	1.5 (0.7, 4.3)
Rectosigmoid junction (21051)	5,296 (0.9%)	103 (1.9%)	18.3 (3.3, 35.2)	19.1%	3.0 (1.4, 7.7)
Rectum (21052)	15,172 (2.5%)	252 (1.7%)	18.0 (5.9, 37.5)	15.8%	2.7 (1.2, 6.7)
Anus, anal canal and anorectum (21060)	2,035 (0.3%)	32 (1.6%)	17.2 (9.2, 36.7)	16.1%	3.3 (1.6, 6.5)
Liver and intrahepatic bile duct					
Liver (21071)	7,473 (1.2%)	76 (1.0%)	12.8 (1.1, 29.4)	53.4%	1.7 (0.8, 5.9)
Intrahepatic bile duct (21072)	2,276 (0.4%)	28 (1.2%)	8.8 (3.1, 15.6)	69.4%	1.3 (0.5, 2.7)
Gallbladder (21080)	1,383 (0.2%)	15 (1.1%)	8.2 (0.6, 14.1)	58.3%	2.4 (1.0, 4.7)
Other biliary (21090)	3,747 (0.6%)	31 (0.8%)	11.7 (3.1, 22.9)	59.4%	1.7 (0.9, 5.2)
Pancreas (21100)	15,220 (2.5%)	137 (0.9%)	4.4 (0.7, 16.0)	70.2%	1.8 (0.9, 4.0)
Retroperitoneum (21110)	489 (0.1%)	8 (1.6%)	33.2 (11.1, 61.2)	28.6%	7.5 (1.2, 28.3)
Peritoneum, omentum, and mesentery (21120)	395 (0.1%)	6 (1.5%)	9.9 (0.5, 16.6)	55.4%	7.2 (3.8, 17.7)
Other digestive organs (21130)	792 (0.1%)	29 (3.7%)	1.2 (0.3, 10.3)	68.2%	2.9 (0.8, 10.8)
Respiratory system					
Lung and bronchus (22030)	77,613 (12.9%)	15,193 (19.6%)	2.1 (0.2, 7.5)	55%	3.8 (1.6, 10.4)
Pleura (22050)	70 (<0.1%)	<6	–	54.3%	–
Trachea, mediastinum, and other respiratoryOrgans (22060)	254 (<0.1%)	25 (9.8%)	3.0 (0.5, 4.6)	50.4%	3.0 (2.0, 5.7)
Skin					
Melanoma of the skin (25010)	24,650 (4.1%)	1,581 (6.4%)*	11.7 (2.1, 26.6)	8.2%	3.4 (1.3, 8.7)
Other nonepithelial skin (25020)	2,613 (0.4%)	47 (1.8%)	8.6 (2.8, 17.8)	15.2%	3.6 (1.1, 12.5)
Breast					
Breast (26000)	87,233 (14.5%)	2,686 (3.1%)*	22.8 (10.9, 40.5)	4.3%	4.6 (1.6, 12.5)
Female genital system					
Cervix uteri (27010)	4,654 (0.8%)	79 (1.7%)	10.9 (5.3, 23.3)	12.1%	3.8 (1.3, 8.9)
Corpus uteri (27020)	19,964 (3.3%)	236 (1.2%)	14.9 (6.5, 32.3)	7.9%	2.5 (1.3, 6.9)
Uterus, NOS (27030)	283 (<0.1%)	9 (3.2%)	10.8 (6.6, 15.9)	56.9%	1.4 (0.9, 2.9)
Ovary (27040)	9,415 (1.6%)	194 (2.1%)	20.5 (13.0, 40.5)	25.5%	4.4 (1.7, 13.5)
Vagina (27050)	632 (0.1%)	20 (3.2%)	7.1 (4.2, 19.9)	30.9%	2.4 (0.8, 3.6)
Vulva (27060)	2,275 (0.4%)	21 (1.0%)	22.2 (6.0, 31.9)	19%	2.9 (1.6, 4.4)
Other female genital organs (27070)	521 (0.1%)	14 (2.7%)	1.5 (0.7, 14.8)	33.2%	8.0 (2.1, 13.0)
Male genital system					
Prostate (28010)	71,335 (11.9%)	473 (0.7%)	21.6 (7.2, 44.8)	4.4%	3.2 (1.3, 12.9)
Testis (28020)	3,471 (0.6%)	45 (1.3%)	5.6 (1.5, 9.0)	1.9%	8.1 (1.9, 17.8)
Penis (28030)	690 (0.1%)	<6	–	20.1%	–
Other male genital organs (28040)	161 (<0.1%)	<6	–	14.3%	–
Urinary system					
Urinary bladder (29010)	16,023 (2.7%)	216 (1.4%)	11.0 (4.6, 20.2)	23.8%	1.9 (1.1, 4.6)
Kidney and renal pelvis (29020)	18,237 (3%)	765 (4.2%)*	7.2 (1.0, 22.0)	16.5%	4.1 (1.6, 11.8)
Ureter (29030)	430 (0.1%)	8 (1.9%)	1.6 (0.0, 14.8)	33.5%	1.5 (0.9, 3.3)
Other urinary organs (29040)	2,450 (0.4%)	45 (1.8%)	15.7 (5.1, 29.6)	25.4%	2.1 (0.9, 4.8)
Endocrine system					
Thyroid (32010)^a^	25,131 (4.2%)	121 (0.5%)	18.1 (5.3, 40.6)	1.7%	7.6 (2.0, 19.2)
Thymus (32020)^c^	506 (0.1%)	12 (2.4%)	6.5 (3.1, 24.9)	11.3%	5.7 (2.4, 10.0)
Adrenal gland (32020)^c^	361 (0.1%)	29 (8.0%)	1.6 (0.5, 4.4)	43.8%	3.5 (1.3, 9.6)
Other endocrine (32020)^b^	115 (<0.1%)	7 (6.1%)	0.3 (0.0, 7.3)	28.7%	45.0 (8.1, 71.7)
Parathyroid gland (32020)^c^	31 (<0.1%)	0 (0%)	–	<10%	–
Lymphoma					
Hodgkin - Nodal (33011)	2,895 (0.5%)	11 (0.4%)	14.2 (4.5, 20.0)	9.3%	27.8 (1.7, 74.0)
Hodgkin - Extranodal (33012)	75 (<0.1%)	<6	–	24%	–
NHL - Nodal (33041)	15,855 (2.6%)	307 (1.9%)	7.8 (3.5, 13.6)	22.7%	3.0 (1.2, 12.9)
NHL - Extranodal (33042)	13,962 (2.3%)	248 (1.9%)	6.7 (2.2, 13.6)	21.3%	3.3 (1.5, 14.3)
Myeloma					
Myeloma (34000)	10,391 (1.7%)	141 (1.4%)	9.2 (2.6, 22.7)	25.6%	3.0 (1.4, 11.8)
Leukemia					
Acute lymphocytic leukemia (35011)	798 (0.1%)	<6	–	34%	–
Chronic lymphocytic leukemia (35012)	6,976 (1.2%)	34 (0.5%)	29.1 (0.9, 56.5)	9.8%	2.2 (1.2, 5.1)
Other lymphocytic leukemia (35013)	796 (0.1%)	8 (1.0%)	4.0 (0.6, 23.0)	14.1%	1.8 (0.7, 3.3)
Acute myeloid leukemia (35021)	4,768 (0.8%)	33 (0.7%)	4.1 (1.4, 11.2)	59.4%	2.4 (1.4, 8.9)
Chronic myeloid leukemia (35022)	2,392 (0.4%)	8 (0.3%)	1.5 (1.0, 3.3)	21.2%	8.2 (5.9, 36.4)
Other myeloid/monocytic leukemia (35023)	156 (<0.1%)	<6	–	57.1%	–
Acute monocytic leukemia (35031)	379 (0.1%)	6 (1.6%)	3.8 (1.8, 9.3)	64.9%	5.4 (2.3, 11.1)
Other acute leukemia (35041)	471 (0.1%)	7 (1.5%)	2.1 (1.5, 13.6)	71.8%	7.1 (3.3, 61.7)
Aleukemic, subleukemic, and NOS (35043)	1,042 (0.2%)	7 (0.7%)	2.6 (0.3, 9.5)	33.2%	1.9 (1.1, 41.2)
Other					
Bones and joints (23000)^b^	1,051 (0.2%)	37 (3.5%)	8.2 (1.9, 15.6)	25.3%	6.9 (2.6, 16.3)
Soft tissue including heart (24000)	4,257 (0.7%)	150 (3.5%)	7.8 (1.9, 17.3)	24.9%	3.4 (1.3, 8.5)
Eye and orbit (30000)	1,166 (0.2%)	32 (2.7%)	17.5 (3.4, 38.1)	6.8%	5.0 (1.3, 20.7)
Mesothelioma (36010)	1,814 (0.3%)	32 (1.8%)	5.6 (1.1, 13.5)	59.6%	1.3 (0.9, 2.8)
Kaposi sarcoma (36020)	333 (0.1%)	<6	–	12.6%	–
Miscellaneous (37000)	25,063 (4.2%)	347 (1.4%)	0.2 (0.0, 3.4)	33.8%	1.9 (0.9, 6.8)
Bone marrow	18,217 (3.0%)	62 (0.3%)	7.5 (1.0, 18.1)	23.4%	2.5 (1.3, 9.7)
Unknown primary site	5,521 (0.9%)	271 (4.9%)	0.0 (0.0, 1.5)	70.0%	1.8 (0.9, 6.0)
Blood	606 (0.1%)	<6	–	14.5%	–
Head, neck, face, not elsewhere classified^b^	287 (<0.1%)	<6	–	23.7%	–
Some lymph node	141 (<0.1%)	<6	–	29.8%	–
Other primary site, not elsewhere classified	291 (<0.1%)	6 (2.1%)	2.0 (0.0, 28.3)	52%	1.2 (1.0, 9.0)
Total	601,678	25,478 (4.2%)	5.2 (0.7, 15.4)	23.2%	3.6 (1.5, 10.1)

^a^Recategorized the SEER recodes 20010–20100, 22010, 22020, or 32010 based on topography as follows:

Lip: C000-C003, C006-C009

Oral cavity: C003-C005, C050, C020-C024, C028-C029, C030-C049, C060-C069, C140-C149

Oropharynx: C010-C019, C051-C059, C090-C099, C100, C102-C109

Salivary glands: C079, C080, C081, C088, C089

Nasopharynx: C11 (same as recode)

Hypopharynx: C129, C130-C139 (same as recode)

Nose and nasal sinus: C300, C301, C310, C311, C313, C318, C319, C312

Larynx: C101, C320-C323, C328, C329

Thyroid: C739 (same as recode)

^b^Brain metastasis only defined using C793 since primary treatment (eg, radiation) may overlap with definition. For SEER recode 23000 if located within the bones of the skull or face and for SEER recode 32020 excluding adrenal gland, parathyroid gland, or thymus gland (predominately the pituitary and pineal glands).

^c^Recategorized as adrenal gland (C749, C740-C741), thymus (C379), and parathyroid gland (<50 cases; C750).

^d^Median time until death or censor.

IQR, interquartile range (25th, 75th percentile); NOS, not otherwise specified; SEER, Surveillance, Epidemiology, and End Results.

### Treatment Indicators of Potential IMD

To identify patients in our cohort with IMD, we first screened the cohort for evidence of treatment that would indicate potential IMD, specifically, cranial irradiation or surgery for resection. Receipt of cranial radiation was identified for 22,322 (3.7%) patients a median 167 (IQR 35, 470) days after primary cancer diagnosis (Figure 1). Surgical resection of a cranial lesion was less common, identified for 3,903 (0.6%) patients a median 277 (IQR 6, 767) days after primary cancer diagnosis (Figure 1).

The most common ICD-10 diagnostic code associated with brain surgery was C793 (“Secondary malignant neoplasm of brain and cerebral meninges”). This was followed by diagnostic codes denoting primary brain tumors, including “benign neoplasms of the cerebral meninges” (D320) or “malignancies of the brain” (C71) (Supplementary Table S3). A review of the pathology reports associated with these resections confirmed the presence of a new primary brain tumor (eg, primary glioblastoma, meningioma, hemangioblastoma, CNS lymphoma), some nonmalignant event (eg, inflammation, hematoma), or non-CNS tumor (eg, facial nerves, skin of the scalp, skull bones).

### Ascertainment of Brain Metastases

We then sought to ascertain if evidence of treatment was a sensible indicator of IMD. Receipt of cranial radiation after the primary cancer diagnosis was associated with worse overall survival (OS), as would be expected as an indicator of IMD (Supplementary Figure S2). However, since many patients with IMD may not receive treatment for IMD, we further explored the prognostic association of the diagnostic code C793 (“Secondary malignant neoplasm of brain and cerebral meninges”). Patients assigned this diagnostic code also had worse OS, supporting the presence of IMD (Supplementary Figure S2). The most common hospital procedure performed in conjunction with the C793 diagnostic code was CT scan of the brain/head (*n* = 3,455; 24%), followed by treatment (excision [11%] or stereotactic surgery [9%]) (Supplementary Table S4). Moreover, as expected, OS was worse among patients with IMD who did not receive treatment versus those who received radiation. Thus, we defined IMD as the first hospital encounter associated with a C793 diagnostic code or cranial radiation, with a few exceptions for specific disease sites. Cranial irradiation was omitted from the definition of IMD for extracranial cancer sites located in the face or head, including carcinomas of the facial or cranial bones, endocrine gland tumors of the pituitary and pineal glands, cancers of the nose and nasal sinus, and miscellaneous tumors of the head, face, or neck. To avoid capturing prophylactic cranial irradiation for acute lymphocytic leukemia, we also relied solely on the C793 code. To avoid capturing new primary CNS disease, we only considered C793 as the definition of IMD for patients who had a subsequent primary malignant or nonmalignant brain cancer diagnosis in the OCR. Using this definition, we identified 25,478 patients with IMD (4.2% of all primary cancers in our cohort), arising a median of 5.2 months (IQR 0.7, 15.4) after primary cancer diagnosis.

### Burden of IMD by Disease Site

**Figure 2.**
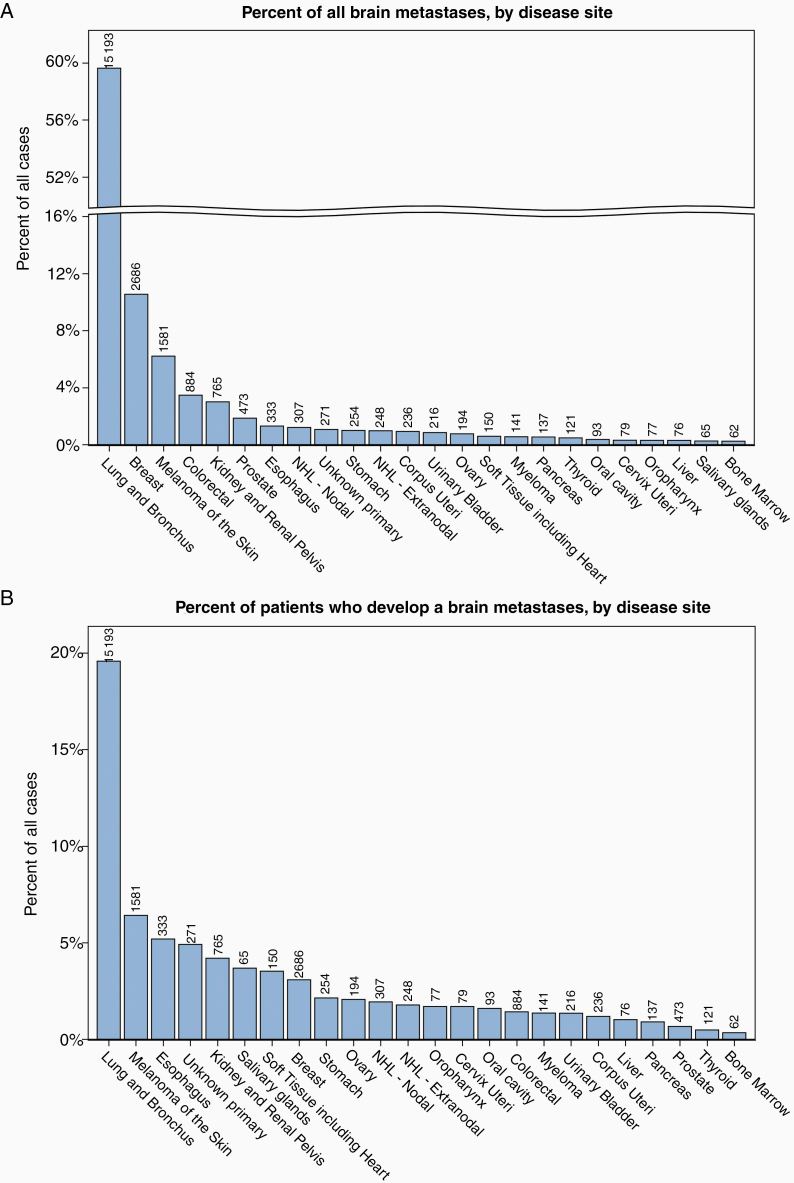

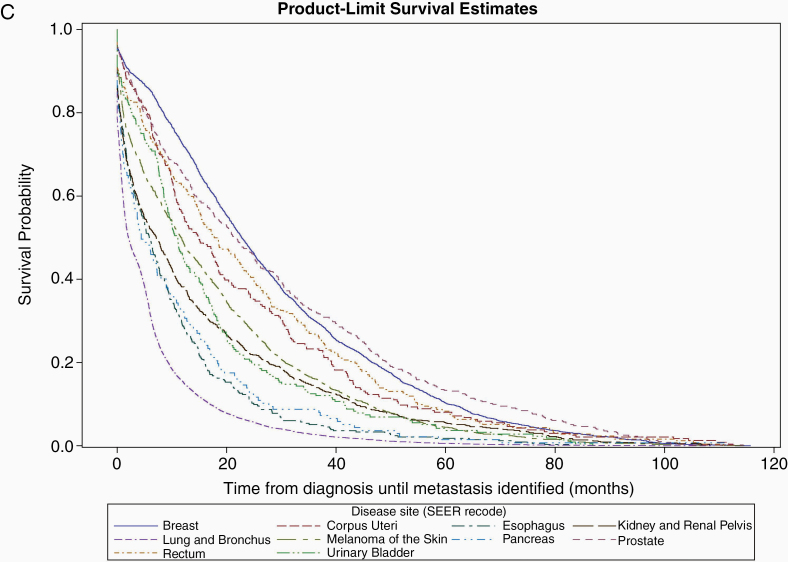
Intracranial metastatic disease by disease site. (A) Percent of all brain metastasis by disease site (minimum 50 metastases). (B) Probability of brain metastasis by disease site (minimum 50 metastases). (C) Time from primary diagnosis until metastasis (select disease sites). SEER, Surveillance, Epidemiology, and End Results.

Lung cancer accounted for the majority of all IMD in our cohort (60%), followed by breast cancer (11%), melanoma (6%), colorectal cancer (4%), and renal cell carcinoma (3%) (Figure 2A; Table 1). The likelihood of developing IMD by primary cancer diagnosis was highest among patients with cancers of the lung and bronchus (20%) (Figure 2B; Table 1).

Among patients with IMD, the time from primary cancer diagnosis until IMD varied by cancer topography (Figure 2C; Table 1). The time to development or identification of IMD was shortest for patients with an unknown primary (*n* = 271; median 1 [IQR 0, 45] days), cancers of the large intestine not otherwise specified (*n* = 78; median 29 IQR [0, 448] days), and cancers of the lung and bronchus (*n* = 15,193; median 62 [IQR 7, 225] days) (Table 1). The time until metastasis was longest for patients with breast cancer (*N* = 2,686; median 688 [IQR 328, 1222] days) and prostate cancer (*N* = 473; median 652 [IQR 216, 1352] days).

We then explored the relationship between 1-year all-cause mortality and the rate of IMD development, by disease site (Table 2; Supplementary Figure S3). The purpose of this qualitative analysis was to present the potential association between the likelihood of IMD, timing of IMD, and 1-year mortality across disease sites. Disease sites having a higher 1-year OS (≥85%, such as breast, prostate, and thyroid) tended to have a lower incidence of IMD (below the average of 4.2% of cases) and a time to IMD longer than the average of 5.2 months. In contrast, patients with melanoma had a higher rate of IMD (6.4%) than other disease sites with similar overall mortality. In these patients, development of IMD had a profound effect on survival: median survival from the time of melanoma diagnosis was 20.4 months for patients who developed IMD; in contrast, median survival was not reached for patients with melanoma without IMD (HR 7.07 [6.62–7.56], *P* < .0001). Patients with a 1-year survival <75%, such as lung and bronchial cancers, also had higher rates of IMD, suggesting that the development of IMD could be one of the main drivers of mortality for patients with these cancers. In contrast, patients with myeloma and cancers of the ovary, liver, and stomach have high mortality rates despite lower incidences of BM, suggesting that these patients are more likely to succumb to progression of systemic disease.

**Table 2.** Relative Odds of Brain Metastasis by Disease Site

Sociodemographic Characteristics	Breast		Lung		Colorectal		Prostate	
	aOR (95% CI)	*P*-value	aOR (95% CI)	*P*-value	aOR (95% CI)	*P*-value	aOR (95% CI)	*P*-value
Age at diagnosis (×10 years)	0.77 (0.74–0.79)	<.0001	0.65 (0.64–0.66)	<.0001	0.85 (0.80–0.89)	<.0001	0.68 (0.60–0.77)	<.0001
Male vs female	0.82 (0.44–1.55)	0.55	0.86 (0.82–0.89)	<.0001	0.96 (0.84–1.10)	0.57	-	-
Rural vs urban^b^	1.07 (0.93–1.25)	0.35	0.96 (0.91–1.02)	0.16	0.78 (0.64–0.96)	0.02	0.66 (0.44–0.98)	0.04
Income quintile^b^								
Highest	1.0 (ref)	0.33	1.0 (ref)	<.0001	1.0 (ref)	0.008	1.0 (ref)	0.82
Mid-high	0.98 (0.85–1.15)		1.01 (0.95–1.08)		1.03 (0.83–1.27)		0.82 (0.56–1.19)	
Middle	0.96 (0.83–1.12)		1.00 (0.93–1.06)		0.83 (0.66–1.03)		0.99 (0.69–1.41)	
Mid-low	0.99 (0.86–1.14)		0.93 (0.87–0.99)		1.17 (0.96–1.413		0.99 (0.69–1.43)	
Lowest	0.87 (0.75–1.01)		0.89 (0.84–0.95)		0.87 (0.70–1.08)		1.01 (0.70–1.46)	
Stage								
I	1.0 (ref)^c^	<.0001	1.0 (ref)	<.0001	1.0 (ref)	<.0001	1.0 (ref)	<.0001
II	2.96 (2.50–3.50)		2.22 (1.98–2.49)		1.33 (0.96–1.85)		1.27 (0.55–2.94)	
III	9.59 (8.09–11.4)		3.29 (3.01–3.59)		3.04 (2.29–4.02)		1.20 (0.48–2.97)	
IV	34.8 (29.1–41.6)		6.32 (5.84–6.84)		6.12 (4.65–8.05)		4.58 (1.85–11.3)	
Unknown	0.77 (0.11–5.56)		1.29 (0.90–1.84)		0.74 (0.30–1.84)		5.04 (0.97–26.3)	
Missing	NR		2.69 (2.45–2.96)		3.84 (2.95–5.19)		NR	
Comorbidity								
Missing	0.94 (0.85–1.04)		1.08 (1.03–1.13)		0.85 (0.72–0.99)		0.81 (0.62–1.06)	
0	1.0 (ref)	0.30	1.0 (ref)	<.0001	1.0 (ref)	0.15	1.0 (ref)	0.09
1	0.92 (0.75–1.12)		0.80 (0.76–0.85)		0.95 (0.77–1.17)		1.09 (0.74–1.60)	
2	0.91 (0.64–1.30)		0.70 (0.65–0.76)		0.76 (0.54–1.08)		0.55 (0.24–1.25)	
3+	0.65 (0.41–1.03)		0.47 (0.43–0.52)		0.78 (0.55–1.12)		0.33 (0.10–1.04)	

^a^Adjusted for age, sex, stage, comorbidity (this table), as well as site-specific factors (Supplementary Table S7), including histology (breast and lung), topography (lung, breast, and colorectal), biomarkers (breast and prostate), and Gleason score (prostate).

^b^Source (or adapted from): Statistics Canada Postal Code Conversion File and Postal Code Conversion File Plus (version 7B, received May 2019) which is based on data licensed from Canada Post Corporation. The patients’ postal code at diagnosis was used.

OR – odds ratio; CI – confidence interval

### Predictors of IMD for Breast, Lung, Colorectal Cancer, and Prostate Cancer

To evaluate whether primary cancers of the same incidence have a statistically higher rate of IMD than the average cancer population, we plotted a funnel plot. The likelihood of developing a brain metastasis was significantly higher for patients with lung cancer of all stages, stage III-IV breast cancer, cancers of the adrenal gland, cancers of the trachea, mediastinum and other respiratory organs, melanoma, and cancers from an unknown primary site (Supplementary Figure S4).

We assessed the relationship of covariates with risk of IMD. The proportion of patients developing IMD stratified by stage (breast, lung, colorectal, and prostate), biomarker status (breast and prostate), and Gleason score (prostate only) is presented in Supplementary Figure S3 and Supplementary Tables S5–S7. Higher stage (*P* < .0001) and younger age (*P* < .0001) were both found to be associated with increased incidence of IMD (Table 2; Supplementary Figure S3; Supplementary Tables S5–S6). Interestingly, patients with greater comorbidity were less likely to develop BM, although this was only statistically significant for patients with lung cancer (*P* < .0001) and trending in a similar direction for the group with the highest comorbidity (3+ vs 0) for breast (OR 0.65 [0.41–1.03]), colorectal (OR 0.78 [0.55–1.12]), and prostate (OR 0.33 [0.10–1.04]).

Breast cancer patients were more likely to develop IMD if they had triple-negative disease (OR 3.52 [3.10–3.99]), HR-/HER2+ disease (OR 2.72 [2.34–3.16]), or HR+/HER2+ disease (OR 1.67 [1.47–1.90]) compared with HR+/HER2- disease. Among patients with HR- disease, triple-negative status was associated with a higher likelihood of IMD overall (OR 1.23 [1.05–1.45]), and among patients with early-stage disease (HR 1.42 [1.09, 1.85], *P* = .01). Ductal carcinomas were more likely to metastasize to the brain than lobular carcinomas (OR 1.44 [1.17–1.78]) even after adjustment for confounding variables. For patients with primary lung cancer, small-cell lung cancers were more commonly associated with IMD (OR 3.45 [3.28–3.63]). Colorectal tumors were least likely to metastasize to the brain if they were located in the appendix and most likely to metastasize to the brain if they were located in the rectosigmoid junction or rectum (*P* < .0001). For patients with prostate cancer, incidence of IMD varied directly with PSA (OR 2.50 [1.76–3.54] if PSA >20 ng/mL) and Gleason score (*P* < .0001).

### Population-Level Estimates

**Figure 3.**
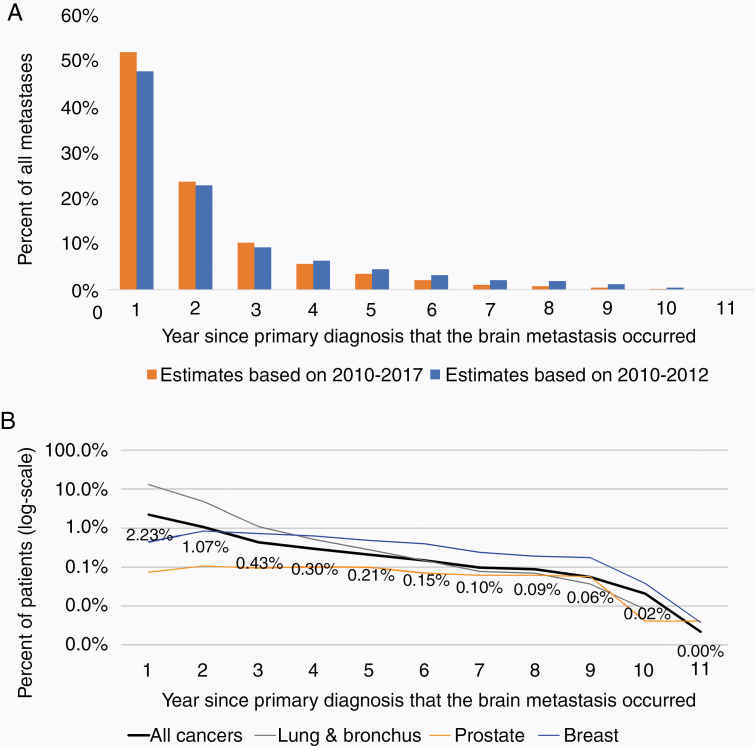
Rate of intracranial metastatic disease (IMD) over time by disease site. (A) Timing of occurrence of brain metastasis since original primary cancer diagnosis. (B) Timing of occurrence of brain metastasis since original primary cancer diagnosis by disease site (logarithmic scale). For a given year, the number of brain metastases is equal to 2.23% of the number of incident cancer diagnosis occurring the previous year plus 1.07% of cases diagnosed the previous year, and so on. Disease-site specific estimates can be derived from the percentages in Supplementary Table S8. To permit sufficient follow-up across disease sites to develop IMD, only primary cancers diagnosed between 2010 and 2012 were included.

Across all primary cancers diagnosed between 2010 and 2017 (to allow for at least 2 years of follow-up), most brain metastases (52%) were identified during the first year after diagnosis (Figure 3A). However, the rates differed by disease site. For example, the likelihood of developing an IMD for lung cancer progressively declined since the time of diagnosis, while for breast cancer patients, the likelihood of IMD was highest during the second and third years after diagnosis (Figure 3B; Supplementary Table S8). Using these incidence estimates, in Ontario, we estimate an annual incidence of 2,894 cases of IMD from primary cancers up to 11 years prior, using an average 65,731 new cancer diagnoses per year. This translates to 19.9 per 100,000 persons for a population of 14.5 million. In comparison, we identified a total 1,916 primary brain tumors diagnosed in 2018 (Supplementary Table S9).

In sensitivity analysis, we used estimates derived from primary cancers diagnosed between 2010 and 2012, allowing at least 7 years of follow-up. This was done because some cancers require up to 5–7 years of follow-up until >90% of IMDs are detected, such as cancers of the breast, prostate, thyroid, anus, and vulva (Supplementary Table S8). Using these estimates only marginally increased the proportion of patients with IMD for select disease sites, such as breast (4.2%) (Supplementary Table S10; Supplementary Figure S5), but increased the overall proportion of patients developing IMD to 4.6%. Using these time-dependent estimates from these patients resulted in 2,842 cases of IMD up to 11 years prior (19.5 per 100,000 persons). However, since the incidence of various cancers has increased over time, we instead applied the time-varying estimates from the 2010–2012 patients to the number of incident diagnoses from 2018, which yielded 3,520 new IMD cases (24.2 per 100,000 persons).

### Brain Metastasis and Mortality

**Figure 4.**
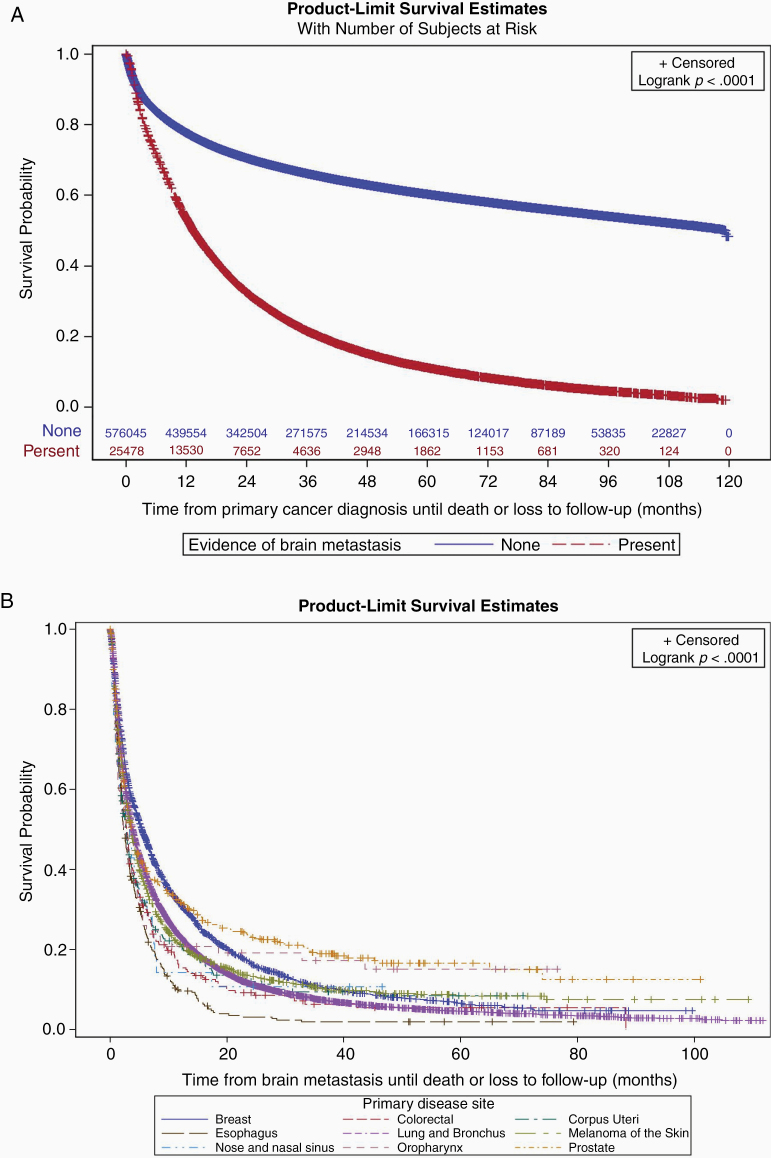
Association of intracranial metastatic disease (IMD) and overall survival. (A) Kaplan–Meier plot for survival in patients with (bottom line) or without (top line) IMD. (B) Kaplan–Meier plot for survival following IMD, by disease site.

IMD was a significant prognostic factor. The median survival across all cancers was 13.4 months for patients who developed IMD, and exceeded 9 years for patients who did not (Figure 4A). The median survival after identification of a brain metastasis was 3.7 (95% CI 3.6, 3.8) months for all cancers. Although there was some variability across disease sites (Table 1), OS was generally poor for patients with most solid tumors who developed IMD (Figure 4B).

## Discussion

Based on the findings of our study, we estimate that approximately 2,842–3,520 patients develop IMD in Ontario every year, arising in 4.2%–4.6% of all patients with a non-CNS cancer. Our study confirms IMD as a substantial cause of CNS disease, with an incidence 5.5× greater than that for glioblastoma, 4× greater than all gliomas, and 1.6× greater than all primary brain tumors combined.

Our finding of an overall rate of IMD of 19.8–24.2 per 100,000 is consistent with previous population-level estimates of 10 and 20 per 100,000 persons.^5^ Further, our study corroborates previous findings regarding primary disease-specific trends, whereby lung, breast, melanoma, kidney, and colorectal cancers account for the majority of all BMs.^10,11,14^ Although these findings in large part reflect the sheer volume of these primary cancers, they also speak to the organotropism of certain primary cancer subgroups towards metastatic spread to the brain.^18,19^

One notable finding of our study was the significant incidence of near synchronous IMD in patients with early stage (stage I–II) lung cancer: 1.9% of patients with early-stage primary lung cancer had evidence of IMD within 6 months of the primary cancer diagnosis. Current guidelines are inconsistent on whether brain imaging should be routinely obtained in this patient population.^20^ Data from Ontario suggest that brain imaging is frequently performed (70%) among early-stage lung cancer patients.^21^ These findings are consistent with practice patterns in other jurisdictions, where some investigators have reported routine brain imaging for lung cancer patients agnostic of stage, and in contradiction to guidelines.^7,8,20,22^ In contrast, for other disease sites like prostate cancer, the risk of brain metastasis is low even among patients with stage III–IV disease.^23^ Our results show that patients with stage I lung cancers have a higher risk of IMD than patients with stage IV prostate cancers (5.7% vs 3.0%), and that IMD arises much sooner after the primary cancer diagnosis in patients with early-stage lung cancer than stage IV prostate cancer (median 14 vs 19 months). Further work is needed to understand the cost-benefit of brain imaging for patients with early-stage lung cancer in the MRI era, but our findings support reflexive brain imaging for at least patients with stage II disease.

Even after accounting for tumor stage and other demographic characteristics, certain histological subtypes (eg, small-cell lung cancers and ductal breast cancers) and tumors with specific biomarkers (eg, triple-negative and HR-/HER2+ breast cancers) were more likely to metastasize to the brain.^7,8,24–26^ Identifying high-risk subpopulations may help inform strategies to promote early detection of IMD. Although there is limited evidence that demonstrates better outcomes with earlier IMD detection, smaller brain metastases are more amenable to disease control and possible cure.^15^ It is conceivable that earlier detection of IMD will result in detection of BMs of smaller size, rendering them more amenable to treatment. Moreover, early detection of IMD may allow for identification of BMs before they become symptomatic, potentially reducing health care costs, improving quality of life, and extending life.^17,27^ The negative implications of screening include increasing patient anxiety and providing treatment with no survival benefit. Among patients with breast cancer, our study agrees with prior reports that IMD is too rare to warrant routine screening, but screening may be justified for patients with metastatic HER2+ or metastatic triple-negative breast cancer.^27,28^ Regardless, breast cancers are more likely to metastasize to other parts of the body before reaching the brain, so extramammary screening in this group should also include standard investigations for lung, liver, and bone metastases.^12^

### Limitations

There are some limitations in our current study. First, it is not always possible to distinguish patients with IMD at the time the primary cancer was diagnosed from patients who developed IMD as a later complication of their primary disease. At least 25% of stage I–III lung cancers had an IMD detected within 6 months of diagnosis, and it is arguable that many of these cases should have been classified as stage IV at the outset. Although the true date of IMD remains uncertain, validation is needed. Despite this, our findings suggest that IMD at diagnosis is more common than cancer registries may observe. Second, we expect some misclassification in brain metastases to occur in both directions (eg, overestimation from false positives; underestimation from false negatives, and IMD stemming from primary cancers occurring >11 years prior). However, we expect the extent of misclassification to be small relative to the true metastasis events reported and any misclassification is unlikely to affect our conclusions. Third, the incidence of IMD in cancers of the face and head may be underestimated since we could not reliably use treatment as an indicator. Despite this, our estimates of brain metastases are low for head and neck cancers, and are consistent with what has been reported in the literature for nose and nasal cavity cancers.^29,30^ Fourth, some of the characteristics that we observed were associated with a lower likelihood of IMD (eg, older age, higher comorbidity) could be explained by competing risks (eg, these are factors associated with worse overall survival) or a decreased tendency to test for the presence of IMD in these patients. Finally, our study does not distinguish patients based on type of IMD (parenchymal, dural-based or leptomeningeal) or number or size of BMs, all of which likely are critical to considerations of disease control and survival.

## Conclusions

We estimate that IMD complicates 4.6% of all patients with a non-CNS cancer in Ontario, affecting up to 24.2 per 100,000 persons. These data demonstrate that IMD should not be considered a rare event. Given the poor survival and quality of life associated with IMD, more accurate, standardized, and timely data collection is needed to direct quality improvement efforts (eg, informing evidence-based guidelines; expanding neuro-oncology expertise; planning for increased availability of stereotactic radiosurgery). Future work should focus on synoptic imaging reports to support and annotate accurate and timely case capture.^31^

## Supplementary Material

vdaa178_suppl_Supplementary_Material
