# Supplementary material for: Incidence and real-world burden of brain metastases from solid tumors and hematologic malignancies in Ontario: a population-based study
Source: Neurooncol Adv. 2020 Dec 22;3(1):vdaa178. doi: 10.1093/noajnl/vdaa178 (PMC7872008; doi:10.1093/noajnl/vdaa178)

# Supplementary Tables

**Table S1:** Administrative codes used to identify brain metastasis

**Table S2:** Surveillance Epidemiology End Result (SEER) recode adjustments to categorize specific cancer types

**Table S3:** International Classification of Disease (ICD, 10^th^ version) list of diagnostic codes associated with brain surgery

**Table S4:** List of intervention codes associated with the International Classification of Disease (ICD, 10^th^ version) diagnostic code C793 (“Secondary malignant neoplasm of brain and cerebral meninges”)

**Table S5:** Factors associated with brain metastasis by disease site

**Table S6:** Brain metastasis and timing by disease site, stage, and biomarker status

**Table S7:** Disease-site-specific factors associated with brain metastasis

**Table S8:** Percent of brain metastasis over time since primary diagnosis, by disease site

**Table S9:** Histological groupings and incidence of primary brain cancers

**Table S10:** Incidence of IMD by disease site, restricted to patients diagnosed between 2010 and 2012

# Supplementary Figures

**Figure S1:** Classification of missing comorbidity information as absence of comorbidity based on overall survival pattern

**Figure S2:** Validation of metastasis definition using overall survival as an outcome indicator. The ICD10 diagnostic code C793 is “Secondary malignant neoplasm of brain and cerebral meninges”.

**Figure S3:** Relationship between tumor aggression and metastasis

**Figure S4:** Probability of IMD by disease site and stage. Funnel plot for the percentage of patients developing a brain metastasis by disease site including all patients in the cohort study period. The upper and lower confidence limits are calculated based on the sample size. Data points falling above (or below) the upper (or lower) limit are disease sites that are statistically significantly more (or less) likely to develop a brain metastasis compared to the overall average (4.3%) for a primary cancer of its incidence.

**Figure S5:** IMD by disease site for primary cancers diagnosed between 2010 and 2012. A) Percent of all brain metastasis by disease site (minimum 50 metastases). B) Probability of brain metastasis by disease site (minimum 50 metastases).

# Table S1: Administrative codes to identify brain metastasis

| **Brain resection** | |
| --- | --- |
| 1AA87 | Excision partial, meninges and dura mater of brain |
| 1AB87 | Excision partial, subarachnoid |
| 1AC87 | Excision partial, ventricles of brain |
| 1AE87 | Excision partial, thalamus and basal ganglia |
| 1AF87 | Excision partial, pituitary region |
| 1AG87 | Excision partial, pineal gland |
| 1AJ87 | Excision partial, cerebellum |
| 1AK87 | Excision partial, cerebellopontine angle |
| 1AN87 | Excision partial, brain |
| 1AP87 | Excision partial, brain stem |
| 1AX87 | Excision partial, meninges |
| 1BA87 | Excision partial, cranial nerves |
| 1EA87 | Excision partial, cranium |
| 1EA92 | Excision partial with reconstruction, cranium |
|  |  |
| **Diagnostic code (ICD10)** | |
| C793 | Secondary malignant neoplasm of brain and cerebral meninges |
| Procedure and diagnostic codes were obtained from DAD (Discharge Abstract Database; includes data on all procures performed in-hospital during an inpatient visit) and NACRS (National Ambulatory Care Reporting System) includes data on all procedures performed in-hospital during an outpatient visit.  ICD10 – International Classification of Disease, 10^th^ revision | |

# Table S2: Surveillance Epidemiology End Result (SEER) Recode adjustments

Head and neck cancers (SEER recodes 20010-20100, 22010, 22020) were reclassified as:

- lip (C000-C003, C006-C009);
- oral cavity (C003-C005, C050, C020-C024, C028-C029, C030-C049, C060-C069, C140-C149);
- oropharynx (C010-C019, C051-C059, C090-C099, C100, C102-C109);
- salivary glands (C079, C080, C081, C088, C089);
- nasopharynx (C11; same as recode);
- hypopharynx (C129, C130-C139; same as recode);
- nose and nasal sinus (C300, C301, C310, C311, C313, C318, C319, C312);
- larynx (C101, C320-C323, C328, C329); and
- thyroid (C739; same as recode).

Miscellaneous cancers (SEER recode 37000) were reclassified as:

- bone marrow (C421, C424);
- unknown primary (C809);
- blood (C420);
- some malignant neoplasm of the head, face, and neck not classified elsewhere (C760, C490, C119, C039, C050, C060, C079, C080, C099, C300, C411, C443, C444);
- some lymph node (C770-C779); and
- miscellaneous (all others).

# Table S3: Diagnostic codes associated with brain surgery

| **Diagnostic code** | **Description** | **N=3,903** |
| --- | --- | --- |
| C793 | Secondary malignant neoplasm of brain and cerebral meninges | 2,809 (72%) |
| D320 | Benign neoplasm of cerebral meninges | 118 (3%) |
| C711 | Malignant neoplasm of frontal lobe | 110 (3%) |
| C712 | Malignant neoplasm of temporal lobe | 94 (2%) |
| C713 | Malignant neoplasm of parietal lobe | 59 (2%) |
| C719 | Malignant neoplasm of brain, unspecified | 30 (1%) |
| C410 | Malignant neoplasm of bones of skull and face | 27 (1%) |
| C795 | Secondary malignant neoplasm of bone and bone marrow | 27 (1%) |
| C833 | Diffuse large B-cell lymphoma | 27 (1%) |
| C714 | Malignant neoplasm of occipital lobe | 25 (1%) |
| G060 | Intracranial and intraspinal abscess and granuloma | 20 (1%) |
| C718 | Malignant neoplasm of overlapping sites of brain | 19 (<1%) |
| I678 | Other specified cerebrovascular diseases | 17 (<1%) |
| T814 | Infection following a procedure | 16 (<1%) |
| C716 | Malignant neoplasm of cerebellum | 15 (<1%) |
| D329 | Benign neoplasm of meninges, unspecified | 13 (<1%) |
| D164 | Benign neoplasm of bones of skull and face | 12 (<1%) |
| D420 | Neoplasm of uncertain behavior of meninges | 11 (<1%) |
| D431 | Neoplasm of uncertain behavior of brain, infratentorial | 11 (<1%) |
| C444 | Other and unspecified malignant neoplasm of skin of scalp and neck | 10 (<1%) |
| C490 | Malignant neoplasm of connective and soft tissue of head, face and neck | 10 (<1%) |
| C700 | Malignant neoplasm of cerebral meninges | 10 (<1%) |
| Intervention codes associated with the ICD-10 diagnostic code C793 (“Secondary malignant neoplasm of brain and cerebral meninges”). All interventions were obtained from the Discharge Abstract Database or the National Ambulatory Care Reporting System. Only interventions with at least 10 observations are shown. | | |

# Table S4: Intervention codes associated with C793

| **Procedure code** | **Description** | **N=14,548** |
| --- | --- | --- |
| 3AN20 | CT scan, brain | 2009 (14%) |
| 1AN87 | Excision partial, brain lobes | 1528 (11%) |
| 3ER20 | CT scan, head | 1446 (10%) |
| 1AN27 | Stereotactic radiosurgery “gamma knife” | 1268 (9%) |
| 3AN40 | MRI, brain | 1050 (7%) |
| 3GY10 | X-ray, chest | 687 (5%) |
| 1AJ87 | Excision partial, cerebellum | 437 (3%) |
| 3ZZ20 | CT scan, whole body | 419 (3%) |
| 3OT20 | CT scan, abdomen | 401 (3%) |
| 1ZZ35 | Chemotherapy, whole body | 275 (2%) |
| 2GT71 | Biopsy, lungs | 236 (2%) |
| 7SF13 | Planning, for radiation therapy treatment | 223 (2%) |
| 3ER40 | MRI, head | 216 (1%) |
| 3GT20 | CT scan, lung and bronchus | 212 (1%) |
| 3SC40 | MRI, spinal vertebrae | 180 (1%) |
| 7SP59 | Education, for nutrition/pre-operative/biofeedback | 180 (1%) |
| 2GM71 | Biopsy, bronchus | 175 (1%) |
| 2ZZ02 | Follow-up with physical exam/naturopathy assessment/activity support | 172 (1%) |
| 1SC27 | Radiation, spinal vertebrae | 151 (1%) |
| Intervention codes associated with the ICD-10 diagnostic code C793 (“Secondary malignant neoplasm of brain and cerebral meninges”). All interventions were obtained from the Discharge Abstract Database or the National Ambulatory Care Reporting System. Only codes present at least 1% of the time are shown.  CT – computed tomography; MRI – magnetic resonance imaging | | |

# Table S5: Factors associated with brain metastasis by disease site using time-to-event analysis

| **Socio-demographic characteristics** | **Breast** | | **Lung** | | **Colorectal** | | | **Prostate** | |
| --- | --- | --- | --- | --- | --- | --- | --- | --- | --- |
|  | **aHR (95% CI)** | **p-value** | **aHR (95% CI)** | **p-value** | | **aHR (95% CI)** | **p-value** | **aHR (95% CI)** | **p-value** |
| Age at diagnosis (x10 years) | 0.83 (0.81-0.86) | <.0001 | 0.78 (0.77-0.79) | <.0001 | | 0.96 (0.91-1.01) | 0.10 | 0.74 (0.65-0.85) | <.0001 |
|  |  |  |  |  | |  |  |  |  |
| Male vs. female | 0.96 (0.53-1.75) | 0.89 | 0.96 (0.93-0.99) | 0.02 | | 0.95 (0.83-1.09) | 0.48 | - | - |
|  |  |  |  |  | |  |  |  |  |
| Rural vs. urban^b^ | 1.09 (0.95-1.25) | 0.23 | 1.00 (0.96-1.05) | 0.96 | | 0.79 (0.64-0.97) | 0.02 | 0.67 (0.45-0.99) | 0.04 |
|  |  |  |  |  | |  |  |  |  |
| Income quintile^b^ |  |  |  |  | |  |  |  |  |
| Highest | 1.0 (ref) | 0.67 | 1.0 (ref) | 0.20 | | 1.0 (ref) | 0.008 | 1.0 (ref) | 0.82 |
| Mid-high | 0.98 (0.85-1.12) |  | 1.04 (0.98-1.10) |  | | 1.04 (0.84-1.28) |  | 0.83 (0.57-1.21) |  |
| Middle | 0.97 (0.85-1.11) |  | 1.02 (0.97-1.07) |  | | 0.84 (0.67-1.05) |  | 1.02 (0.71-1.45) |  |
| Mid-low | 1.02 (0.89-1.16) |  | 0.99 (0.94-1.05) |  | | 1.21 (0.99-1.48) |  | 1.01 (0.71-1.45) |  |
| Lowest | 0.92 (0.80-1.06) |  | 0.98 (0.92-1.03) |  | | 0.93 (0.75-1.15) |  | 1.03 (0.72-1.49) |  |
|  |  |  |  |  | |  |  |  |  |
| Stage |  |  |  |  | |  |  |  |  |
| I | 1.0 (ref) | <.0001 | 1.0 (ref) | <.0001 | | 1.0 (ref) | <.0001 | 1.0 (ref) | <.0001 |
| II | 3.07 (2.59-3.63) |  | 2.44 (2.20-2.71) |  | | 1.41 (1.01-1.95) |  | 1.15 (0.50-2.64) |  |
| III | 10.7 (9.06-12.7) |  | 4.57 (4.20-4.96) |  | | 3.62 (2.74-4.79) |  | 1.08 (0.44-2.66) |  |
| IV | 58.5 (49.3-69.5) |  | 12.3 (11.4-13.3) |  | | 16.6 (12.6-21.9) |  | 5.51 (2.25-13.5) |  |
| Unknown | 0.84 (0.12-6.01) |  | 4.53 (4.14-4.96) |  | | 7.91 (5.84-10.7) |  | 5.06 (1.00-25.6) |  |
| Missing | NR |  | 2.08 (1.48-2.93) |  | | 1.31 (0.53-3.27) |  | NR |  |
|  |  |  |  |  | |  |  |  |  |
| Comorbidity |  |  |  |  | |  |  |  |  |
| Missing | 0.91 (0.83-1.00) |  | 1.06 (1.02-1.10) |  | | 0.78 (0.67-0.91) |  | 0.76 (0.58-0.99) |  |
| 0 | 1.0 (ref) | 0.25 | 1.0 (ref) | <.0001 | | 1.0 (ref) | 0.02 | 1.0 (ref) | 0.06 |
| 1 | 1.03 (0.86-1.24) |  | 0.88 (0.84-0.93) |  | | 1.05 (0.85-1.29) |  | 1.15 (0.79-1.69) |  |
| 2 | 1.12 (0.79-1.57) |  | 0.81 (0.76-0.87) |  | | 0.91 (0.64-1.28) |  | 0.60 (0.26-1.36) |  |
| 3+ | 0.84 (0.55-1.31) |  | 0.61 (0.56-0.67) |  | | 1.11 (0.78-1.58) |  | 0.41 (0.13-1.28) |  |
| ^a^ adjusted for age, sex, stage, comorbidity (this table), as well as site-specific factors (Table S7), including histology (breast and lung), topography (lung, breast, and colorectal), biomarkers (breast and prostate), and Gleason score (prostate)  ^b^ source (or adapted from): Statistics Canada Postal Code Conversion File and Postal Code Conversion File Plus (version 7B, received May 2019) which is based on data licensed from Canada Post Corporation. The patients’ postal code at diagnosis was used.  OR – odds ratio; CI – confidence interval; NR – not reportable (too few events) | | | | | | | | | |

# Table S6: Brain metastasis and timing by disease site, stage, and biomarker status

| Disease site (SEER recode) | N (%) with brain metastases | N Total | Median (IQR) time until metastasis (months) |
| --- | --- | --- | --- |
| Lung and Bronchus (22030) |  |  |  |
| Stage I | 755 (5.7%) | 13,227 | 13.9 (6.0, 26.6) |
| Stage II | 627 (12.0%) | 5,230 | 10.5 (6.0, 21.3) |
| Stage III | 2,466 (18.9%) | 13,073 | 7.2 (5.0, 13.4) |
| Stage IV | 9,950 (28.9%) | 34,389 | 1.0 (0.0, 4.8) |
| Unknown stage | 35 (5.4%) | 650 | 6.2 (4.3, 10.0) |
| Missing stage | 1,360 (12.3%) | 11,044 | 1.3 (0.0, 6.8) |
|  |  |  |  |
| Breast (26000) |  |  |  |
| Stage I-II, ER-, PR-, HER2- | 229 (4.5%) | 5,061 | 25.0 (16.1, 39.1) |
| Stage I-II, ER-, PR-, HER2+ | 77 (3.2%) | 2,425 | 25.1 (16.4, 39.9) |
| Stage I | 220 (0.6%) | 36,958 | 38.7 (18.1, 58.2) |
| Stage II | 624 (2.2%) | 28,622 | 30.1 (18.3, 51.0) |
| Stage III | 708 (7.1%) | 10,044 | 25.4 (14.8, 43,6) |
| Stage IV | 724 (17.7%) | 4,102 | 14.5 (3.4, 27.2) |
| Unknown stage | 358 (5.3%) | 6,732 | 16.5 (4.0, 33.8) |
| Missing stage | 45 (11.6%) | 389 | 9.9 (5.4, 19.2) |
|  |  |  |  |
| Prostate (28010) |  |  |  |
| Stage I | 39 (0.3%) | 15,251 | 44.0 (15.2, 65.5) |
| Stage II | 134 (0.4%) | 33,442 | 25.0 (6.2, 47.8) |
| Stage III | 40 (0.4%) | 9,351 | 41.7 (13.0, 68.0) |
| Stage IV | 220 (3.0%) | 7,312 | 18.7 (0.7, 35.0) |
| Unknown/missing stage | 40 (0.7%) | 5,979 | 17.2 (4.2, 32.5) |
| PSA <10ng/mL | 95 (0.3%) | 29,833 | 30.4 (6.5, 62.7) |
| PSA 10-20 ng/mL | 42 (0.5%) | 9,396 | 33.8 (9.0, 62.9) |
| PSA >20ng/mL | 182 (2.3%) | 8,075 | 22.0 (9.3, 42.1) |
| Gleason 6 | 38 (0.2%) | 17,088 | 47.3 (17.2, 76.6) |
| Gleason 7 | 103 (0.4%) | 24,211 | 20.9 (5.3, 54.6) |
| Gleason 8 | 52 (1.0%) | 5,132 | 26.4 (13.8, 50.5) |
| Gleason 9 | 114 (1.9%) | 5,908 | 29.6 (13.4, 43.9) |
| Gleason 10 | 16 (2.7%) | 594 | 19.0 (11.6, 45.1) |
|  |  |  |  |
| Colorectal (21041-21052) |  |  |  |
| Stage I | 62 (0.6%) | 12,966 | 28.2 (11.7, 48.5) |
| Stage II | 85 (0.6%) | 14,230 | 32.7 (11.6, 43.9) |
| Stage III | 244 (1.5%) | 16,140 | 30.5 (16.3, 47.4) |
| Stage IV | 338 (3.0%) | 11,206 | 6.4 (0.6, 23.5) |
| Unknown/missing stage | 158 (1.7%) | 9,176 | 9.1 (1.0, 17.7) |
|  |  |  |  |

# Table S7: Disease-site-specific factors associated with brain metastasis

| **Site-specific factors** |  | **Odds of developing a brain metastasis** | | **Time-to-event analysis** | |
| --- | --- | --- | --- | --- | --- |
|  | **N (%)** | **Odds ratio (95% CI)^a^** | **p-value** | **Hazard ratio (95% CI)^a,b^** | **p-value** |
| **Breast cancer** |  |  |  |  |  |
| Histology |  |  |  |  |  |
| Lobular | 7,080 (8%) | 1.0 (ref) | 0.002 | 1.0 (ref) | 0.0008 |
| Ductal | 76,596 (88%) | 1.44 (1.16-1.78) |  | 1.47 (1.20-1.81) |  |
| Ductal and lobular | 3,557 (4%) | 1.64 (1.22-2.22) |  | 1.58 (1.18-2.10) |  |
|  |  |  |  |  |  |
| Topography |  |  |  |  |  |
| Upper-outer quadrant | 31,279 (36%) | 1.0 (ref) | 0.83 | 1.0 (ref) | 0.99 |
| Axillary tail of breast | 520 (1%) | 0.83 (0.48-1.44) |  | 0.92 (0.55-1.54) |  |
| Breast, NOS | 9,060 (10%) | 0.88 (0.73-1.05) |  | 1.02 (0.87-1.21) |  |
| Central portion of breast | 4,890 (6%) | 1.04 (0.85-1.27) |  | 1.00 (0.83-1.21) |  |
| Lower-inner quadrant | 5,179 (6%) | 1.04 (0.84-1.29) |  | 1.00 (0.82-1.21) |  |
| Lower-outer quadrant | 8,206 (9%) | 1.07 (0.90-1.26) |  | 1.05 (0.90-1.23) |  |
| Nipple | 1,785 (2%) | 0.96 (0.66-1.39) |  | 0.94 (0.67-1.33) |  |
| Overlapping lesion | 14,849 (17%) | 1.02 (0.89-1.16) |  | 1.06 (0.94-1.21) |  |
| Upper-inner quadrant | 11,465 (13%) | 1.02 (0.87-1.19) |  | 0.99 (0.86-1.16) |  |
|  |  |  |  |  |  |
| Biomarkers |  |  |  |  |  |
| HR+, HER2- | 45,678 (70%) | 1.0 (ref) | <.0001 | 1.0 (ref) | <.0001 |
| HR+, HER2+ | 9,029 (14%) | 1.67 (1.47-1.90) |  | 1.58 (1.39-1.78) |  |
| HR-, HER2+ | 3,592 (6%) | 2.72 (2.34-3.16) |  | 2.61 (2.27-3.00) |  |
| HR-, HER2- (triple-negative) | 6,472 (10%) | 3.52 (3.10-3.99) |  | 4.28 (3.81-4.81) |  |
|  |  |  |  |  |  |
| **Lung cancer** |  |  |  |  |  |
| Histology |  |  |  |  |  |
| Non-small-cell carcinoma | 69,355 (89%) | 1.0 (ref) | <.0001 | 1.0 (ref) | <.0001 |
| Small-cell carcinoma | 8,258 (11%) | 3.45 (3.28-3.63) |  | 2.70 (2.60-2.81) |  |
|  |  |  |  |  |  |
| Topography |  |  |  |  |  |
| Upper lobe | 38,805 (50%) | 1.0 (ref) | <.0001 | 1.0 (ref) | <.0001 |
| Lower lobe | 19,969 (26%) | 0.92 (0.88-0.97) |  | 0.93 (0.89-0.97) |  |
| Lung, NOS | 10,932 (14%) | 0.51 (0.48-0.54) |  | 0.78 (0.74-0.82) |  |
| Main bronchus | 4,098 (5%) | 0.74 (0.68-0.80) |  | 0.90 (0.85-0.96) |  |
| Middle lobe | 2,406 (4%) | 0.90 (0.82-0.99) |  | 0.90 (0.83-0.98) |  |
| Overlapping lesion of lung | 403 (1%) | 0.68 (0.52-0.88) |  | 0.95 (0.76-1.18) |  |
|  |  |  |  |  |  |
| **Colorectal cancer** |  |  |  |  |  |
| Topography |  |  |  |  |  |
| Transverse colon | 3,814 (6%) | 1.0 (ref) | <.0001 | 1.0 (ref) | <.0001 |
| Appendix | 1,778 (3%) | 0.15 (0.05-0.48) |  | 0.12 (0.04-0.38) |  |
| Ascending colon | 8,378 (13%) | 1.69 (1.13-2.52) |  | 1.51 (1.01-2.25) |  |
| Cecum | 10,302 (16%) | 1.46 (0.99-2.17) |  | 1.38 (0.93-2.04) |  |
| Descending colon | 2,479 (4%) | 1.53 (0.93-2.50) |  | 1.27 (0.77-2.09) |  |
| Hepatic flexure | 1,941 (3%) | 1.02 (0.56-1.85) |  | 0.92 (0.50-1.70) |  |
| Large intestine, NOS | 2,093 (3%) | 3.10 (2.03-4.75) |  | 3.90 (2.55-5.96) |  |
| Rectosigmoid junction | 5,296 (8%) | 1.96 (1.31-2.95) |  | 1.68 (1.12-2.52) |  |
| Rectum | 15,172 (24%) | 1.87 (1.28-2.73) |  | 1.69 (1.16-2.45) |  |
| Sigmoid colon | 11,415 (18%) | 1.24 (0.84-1.84) |  | 1.06 (0.71-1.57) |  |
| Splenic flexure | 1,345 (2%) | 1.22 (0.662.28) |  | 1.04 (0.55-1.95) |  |
|  |  |  |  |  |  |
| **Prostate cancer** |  |  |  |  |  |
| Prostate-specific antigen |  |  |  |  |  |
| <10 ng/mL | 29,833 (63%) | 1.0 (ref) | <.0001 | 1.0 (ref) | <.0001 |
| 10-20 ng/mL | 9,396 (20%) | 1.17 (0.80-1.72) |  | 1.20 (0.82-1.76) |  |
| >20 ng/mL | 8.075 (17%) | 2.50 (1.76-3.54) |  | 2.83 (2.01-3.98) |  |
|  |  |  |  |  |  |
| Gleason score |  |  |  |  |  |
| 6 | 17,088 (32%) | 1.0 (ref) | <.0001 | 1.0 (ref) | <.0001 |
| 7 | 24,211 (46%) | 2.00 (1.03-3.90) |  | 2.19 (1.13-4.24) |  |
| 8 | 5,132 (10%) | 3.14 (1.53-6.44) |  | 3.46 (1.70-7.05) |  |
| 9 | 5,908 (11%) | 4.16 (2.06-8.40) |  | 5.20 (2.59-10.4) |  |
| 10 | 594 (1%) | 4.91 (2.07-11.7) |  | 7.63 (3.26-17.8) |  |
| ^a^ adjusted for age, sex, stage, comorbidity, rurality, and income quintile (Table 2), as well as site-specific factors (this table), including histology (breast and lung), topography (lung, breast, and colorectal), biomarkers (breast and prostate), and Gleason score (prostate)  ^b^ censored at death or last contact date  OR – odds ratio; CI – confidence interval; NOS – not otherwise specified; HR – hormone receptor (negative if both estrogen and progesterone receptors are negative; positive if either estrogen or progesterone receptor is positive); HER2 – human epidermal grown factor-2 | | | | | |

# Table S8: Percent of brain metastasis over time since primary diagnosis, by disease site

|  | **N primary diagnoses in 2018** | **Brain metastasis occurred within the n^th^ year after diagnosis for patients diagnosed between 2010 and 2012** | | | | | | | | | | |
| --- | --- | --- | --- | --- | --- | --- | --- | --- | --- | --- | --- | --- |
|  |  | **1st** | **2nd** | **3rd** | **4th** | **5th** | **6th** | **7th** | **8^th^** | **9^th^** | **10^th^** | **11^th^** |
| **Total** | **75829** | **2.23%** | **1.07%** | **0.43%** | **0.30%** | **0.21%** | **0.15%** | **0.10%** | **0.09%** | **0.06%** | **0.02%** | **-** |
| Lung and Bronchus | 9136 | 12.99% | 4.72% | 1.09% | 0.51% | 0.28% | 0.15% | 0.08% | 0.07% | 0.04% | - | - |
| Breast | 11143 | 0.45% | 0.85% | 0.73% | 0.63% | 0.48% | 0.40% | 0.24% | 0.19% | 0.18% | 0.04% | - |
| Melanoma of the Skin | 3705 | 2.17% | 1.73% | 1.50% | 0.89% | 0.74% | 0.48% | 0.32% | 0.20% | - | - | - |
| Kidney and Renal Pelvis | 2384 | 2.38% | 1.04% | 0.41% | 0.62% | 0.26% | - | 0.19% | 0.23% | - | - | - |
| Prostate | 9329 | 0.07% | 0.11% | 0.09% | 0.10% | 0.10% | 0.07% | 0.06% | 0.06% | 0.06% | - | - |
| Miscellaneous | 2999 | 0.92% | 0.15% | - | - | - | - | - | - | - | - | - |
| NHL - Nodal | 2038 | 1.10% | 0.97% | 0.17% | - | - | - | - | - | - | - | - |
| Esophagus | 791 | 2.35% | 1.63% | 0.53% | - | - | - | - | - | - | - | - |
| NHL - Extranodal | 1768 | 0.55% | 0.61% | 0.17% | - | - | - | - | - | - | - | - |
| Corpus Uteri | 2754 | 0.19% | 0.41% | 0.24% | 0.14% | 0.12% | 0.10% | - | - | - | - | - |
| Colorectal | 7315 | 0.33% | 0.27% | 0.28% | 0.26% | 0.16% | 0.16% | 0.06% | 0.08% | 0.06% | - | - |
| Stomach | 1464 | 0.83% | 0.64% | 0.25% | 0.25% | - | - | - | - | - | - | - |
| Urinary Bladder | 1915 | 0.30% | 0.61% | 0.20% | 0.14% | - | - | - | - | - | - | - |
| Acute Lymphocytic Leukemia | 106 | - | - | - | - | - | - | - | - | - | - | - |
| Ovary | 1134 | 0.31% | 0.45% | - | 0.34% | 0.38% | 0.27% | - | - | - | - | - |
| Thyroid | 2927 | 0.10% | 0.09% | - | 0.08% | - | - | - | - | - | - | - |
| Soft Tissue including Heart | 625 | 1.45% | 1.36% | - | - | - | - | - | - | - | - | - |
| Myeloma | 1389 | 0.62% | 0.31% | - | - | - | - | - | - | - | - | - |
| Pancreas | 1546 | 0.38% | 0.13% | - | - | - | - | - | - | - | - | - |
| Oral cavity | 817 | 0.49% | 0.43% | 0.43% | - | - | - | - | - | - | - | - |
| Bones and Joints | 170 | - | - | - | - | - | - | - | - | - | - | - |
| Nose and nasal sinus | 113 | - | - | - | - | - | - | - | - | - | - | - |
| Salivary Glands | 239 | - | - | - | - | - | - | - | - | - | - | - |
| Other Endocrine including Thymus | 11 | - | - | - | - | - | - | - | - | - | - | - |
| Cervix Uteri | 606 | 0.38% | 0.64% | - | - | - | - | - | - | - | - | - |
| Liver | 881 | - | 0.32% | - | - | - | - | - | - | - | - | - |
| Other Non-Epithelial Skin | 449 | 0.94% | - | - | - | - | - | - | - | - | - | - |
| Adrenal gland | 49 | 6.42% | - | - | - | - | - | - | - | - | - | - |
| Chronic Lymphocytic Leukemia | 886 | 0.30% | - | - | - | - | - | - | - | - | - | - |
| Nasopharynx | 96 | - | - | - | - | - | - | - | - | - | - | - |
| Other Urinary Organs | 349 | - | - | - | - | - | - | - | - | - | - | - |
| Thymus | 67 | - | - | - | - | - | - | - | - | - | - | - |
| Testis | 481 | 0.57% | - | - | - | - | - | - | - | - | - | - |
| Eye and Orbit | 153 | - | - | - | - | - | - | - | - | - | - | - |
| Acute Myeloid Leukemia | 598 | 0.57% | - | - | - | - | - | - | - | - | - | - |
| Small Intestine | 515 | 0.63% | - | - | - | - | - | - | - | - | - | - |
| Parathyroid | 9 | - | - | - | - | - | - | - | - | - | - | - |
| Other Biliary | 563 | - | - | - | - | - | - | - | - | - | - | - |
| Mesothelioma | 178 | - | - | - | - | - | - | - | - | - | - | - |
| Anus, Anal Canal and Anorectum | 341 | - | - | - | - | - | - | - | - | - | - | - |
| Larynx | 375 | - | - | - | - | - | - | - | - | - | - | - |
| Other Digestive Organs | 95 | - | - | - | - | - | - | - | - | - | - | - |
| Intrahepatic Bile Duct | 251 | - | - | - | - | - | - | - | - | - | - | - |
| Trachea, Mediastinum and Other Respiratory Organs | 29 | 8.42% | - | - | - | - | - | - | - | - | - | - |
| Vulva | 310 | - | - | - | - | - | - | - | - | - | - | - |
| Vagina | 94 | - | - | - | - | - | - | - | - | - | - | - |
| Hypopharynx | 62 | - | - | - | - | - | - | - | - | - | - | - |
| Other Female Genital Organs | 103 | - | - | - | - | - | - | - | - | - | - | - |
| Chronic Myeloid Leukemia | 289 | - | - | - | - | - | - | - | - | - | - | - |
| Gallbladder | 168 | - | - | - | - | - | - | - | - | - | - | - |
| Hodgkin - Nodal | 335 | - | - | - | - | - | - | - | - | - | - | - |
| Uterus, NOS | 26 | - | - | - | - | - | - | - | - | - | - | - |
| Oropharynx | 624 | - | 0.51% | - | - | - | - | - | - | - | - | - |
| Ureter | 54 | - | - | - | - | - | - | - | - | - | - | - |
| Aleukemic, subleukemic and NOS | 109 | - | - | - | - | - | - | - | - | - | - | - |
| Other Lymphocytic Leukemia | 99 | - | - | - | - | - | - | - | - | - | - | - |
| Other Acute Leukemia | 43 | - | - | - | - | - | - | - | - | - | - | - |
| Lip | 96 | - | - | - | - | - | - | - | - | - | - | - |
| Retroperitoneum | 80 | - | - | - | - | - | - | - | - | - | - | - |
| Penis | 101 | - | - | - | - | - | - | - | - | - | - | - |
| Acute Monocytic Leukemia | 19 | - | - | - | - | - | - | - | - | - | - | - |
| Peritoneum, Omentum and Mesentery | 40 | - | - | - | - | - | - | - | - | - | - | - |
| Appendix | 275 | - | - | - | - | - | - | - | - | - | - | - |
| Pleura | 14 | - | - | - | - | - | - | - | - | - | - | - |
| Other Male Genital Organs | 26 | - | - | - | - | - | - | - | - | - | - | - |
| Kaposi Sarcoma | 38 | - | - | - | - | - | - | - | - | - | - | - |
| Hodgkin - Extranodal | 17 | - | - | - | - | - | - | - | - | - | - | - |
| Other Myeloid/Monocytic Leukemia | 18 | - | - | - | - | - | - | - | - | - | - | - |
| Disease Sites with <6 brain metastasis cases are indicated by a hyphen (-) | | | | | | | | | | | | |

# Table S9: Histological groupings

| **ICD histology code** | **Description** |
| --- | --- |
| **High-grade glioma** |  |
| 9380 | Glioma, malignant |
| 9381 | Gliomatosis cerebri |
| 9401 | Astrocytoma, anaplastic, NOS |
| 9440 | Glioblastoma, NOS |
| 9441 | Giant cell glioblastoma |
| 9442 | Gliosarcoma/gliofibroma |
| 9505 | Ganglioglioma, anaplastic or NOS |
| **Subtotal** | **664** |
|  |  |
| **Glioblastoma** |  |
| 9440-9442 | Glioblastoma |
| **Subtotal** | **549** |
|  |  |
| **Low-grade glioma** |  |
| 9451 | Oligodendroglioma, anaplastic, NOS |
| 9400 | Astrocytoma, NOS |
| 9450 | Oligodendroglioma, NOS |
| 9382 | Oligoastrocytoma, NOS |
| 9420 | Fibrillary astrocytoma |
| 9411 | Gemistocytic astrocytoma, NOS |
| 9384 | Subependymal giant cell astrocytoma |
| **Subtotal** | **129** |
|  |  |
| **Meningioma** |  |
| 9530 | Meningioma, NOS |
| 9539 | Atypical meningioma |
| 9531 | Meningothelial meningioma |
| 9537 | Transitional meningioma |
| 9532 | Fibrous meningioma |
| 9533 | Psammomatous meningioma |
| 9534 | Angiomatous meningioma |
| 9530 | Meningioma, malignant |
| 9538 | Clear cell meningioma |
| 8815 | Hemangiopericytoma, malignant or NOS |
| 9538 | Papillary meningioma |
| 9539 | Meningeal sarcomatosis |
| 8728 | Meningeal melanocytoma, melanocytosis, or melanomatosis |
| **Subtotal** | **647** |
|  |  |
| **Ependymoma** |  |
| 9391 | Ependymoma, NOS |
| 9394 | Myxopapillary ependymoma |
| 9383 | Subependymoma |
| 9392 | Ependymoma, anaplastic |
| 9391 | Sellar ependymoma |
| **Subtotal** | **43** |
|  |  |
| **Lymphoma** |  |
| 9680 | Diffuse large B-cell lymphoma, NOS |
| 9591 | Malignant lymphoma, non-Hodgkin |
| 9590 | Malignant lymphoma, NOS |
| 9699 | Marginal zone B-cell lymphoma, NOS |
| 9712 | Intravascular large B-cell lymphoma |
| 9702 | Mature T-cell lymphoma, NOS |
| 9698 | Follicular lymphoma, grade 3 |
| 9691 | Follicular lymphoma, grade 2 |
| 9687 | Burkitt lymphoma, NOS |
| 9663 | Hodgkin lymphoma, nodular sclerosis, NOS |
| 9673 | Mantle cell lymphoma |
| 9714 | Anaplastic large cell lymphoma, T-cell and Null-cell type |
| 9823 | B-cell chronic lymphocytic leukemia/small lymphocytic lymphoma |
| 9688 | T-cell/histiocyte rich large B-cell lymphoma |
| 9827 | Adult T-cell leukemia/lymphoma (HTLV-1 positive) |
| 9811 | B lymphoblastic leukemia/lymphoma, NOS |
| 9695 | Follicular lymphoma, grade 1 |
| 9652 | Hodgkin lymphoma, mixed cellularity, NOS |
| 9671 | Lymphoplasmacytic lymphoma |
| **Subtotal** | **88** |
|  |  |
| **Medulloblastoma** |  |
| 9470 | Medulloblastoma, NOS |
| 9473 | CNS embryonal tumor, NOS |
| 9471 | Desmoplastic nodular medulloblastoma |
| 9474 | Large cell medulloblastoma |
| 9508 | Atypical teratoid/rhabdoid tumor |
| **Subtotal** | **7** |
|  |  |
| **Schwannoma** |  |
| 9560 | Schwannoma, NOS |
| **Subtotal** | **232** |
|  |  |
| **Pilocytic astrocytoma** | |
| 9421 | Pilocytic astrocytoma |
| **Subtotal** | **16** |
|  |  |
| **Other** | |
| C700-C729 | Any other histology |
| **Subtotal** | **90** |
| **Total** | **1,916** |

# Table S10: Incidence of IMD by disease site, restricted to patients diagnosed between 2010 and 2012

| **Primary disease site** | **Number with IMD** | **Percentage with IMD** |  |
| --- | --- | --- | --- |
| **Head and neck** |  |  |  |
| Salivary glands^a^ | 13 | 2.8% |  |
| Nasopharynx (20060)^a^ | 12 | 4.7% |  |
| Nose and nasal sinus^a,b^ | 9 | 4.0% |  |
| Oropharynx (20080)^a^ | 23 | 2.0% |  |
| Oral cavity^a^ | 29 | 1.8% |  |
| Larynx^a^ | 10 | 1.1% |  |
|  |  |  |  |
| **Digestive system** |  |  |  |
| Esophagus (21010) | 107 | 5.1% |  |
| Stomach (21020) | 78 | 2.2% |  |
| Small Intestine (21030) | 12 | 1.3% |  |
| Cecum (21041) | 44 | 1.3% |  |
| Appendix (21042) | 0 | 0% |  |
| Ascending Colon (21043) | 43 | 1.7% |  |
| Hepatic Flexure (21044) | 7 | 1.1% |  |
| Transverse Colon (21045) | 15 | 1.3% |  |
| Splenic Flexure (21046) | 10 | 2.5% |  |
| Descending Colon (21047) | 12 | 1.5% |  |
| Sigmoid Colon (21048) | 48 | 1.3% |  |
| Large Intestine, NOS (21049) | 12 | 1.8% |  |
| Rectosigmoid Junction (21051) | 47 | 2.4% |  |
| Rectum (21052) | 92 | 2.0% |  |
| Anus, Anal Canal and Anorectum (21060) | 9 | 1.6% |  |
|  |  |  |  |
| **Liver and intrahepatic bile duct** |  |  |  |
| Liver (21071) | 27 | 1.3% |  |
| Intrahepatic Bile Duct (21072) | 8 | 1.2% |  |
| Other Biliary (21090) | 9 | 0.9% |  |
| Pancreas (21100) | 33 | 0.7% |  |
| Other Digestive Organs (21130) | 6 | 2.5% |  |
|  |  |  |  |
| **Respiratory system** |  |  |  |
| Lung and Bronchus (22030) | 4881 | 19.9% |  |
| Trachea, mediastinum, & other respiratory Organs (22060) | 11 | 11.6% |  |
|  |  |  |  |
| **Skin** |  |  |  |
| Melanoma of the Skin (25010) | 531 | 8.2% |  |
| Other Non-Epithelial Skin (25020) | 12 | 1.6% |  |
|  |  |  |  |
| **Breast** |  |  |  |
| Breast (26000) | 1098 | 4.2% |  |
|  |  |  |  |
| **Female genital system** |  |  |  |
| Cervix Uteri (27010) | 27 | 1.7% |  |
| Corpus Uteri (27020) | 84 | 1.4% |  |
| Ovary (27040) | 65 | 2.2% |  |
| Vagina (27050) | 8 | 4.7% |  |
| Vulva (27060) | 10 | 1.7% |  |
|  |  |  |  |
| **Male genital system** |  |  |  |
| Prostate (28010) | 179 | 0.7% |  |
| Testis (28020) | 12 | 1.1% |  |
|  |  |  |  |
| **Urinary system** |  |  |  |
| Urinary Bladder (29010) | 73 | 1.5% |  |
| Kidney and Renal Pelvis (29020) | 284 | 5.4% |  |
| Other Urinary Organs (29040) | 9 | 1.4% |  |
|  |  |  |  |
| **Endocrine system** |  |  |  |
| Thyroid (32010)^a^ | 41 | 0.5% |  |
| Adrenal gland (32020)^c^ | 8 | 7.3% |  |
|  |  |  |  |
| **Lymphoma** |  |  |  |
| NHL - Nodal (33041) | 124 | 2.6% |  |
| NHL - Extranodal (33042) | 58 | 1.7% |  |
|  |  |  |  |
| **Myeloma** |  |  |  |
| Myeloma (34000) | 43 | 1.5% |  |
|  |  |  |  |
| **Leukemia** |  |  |  |
| Chronic Lymphocytic Leukemia (35012) | 13 | 0.7% |  |
| Acute Myeloid Leukemia (35021) | 12 | 0.9% |  |
|  |  |  |  |
| **Other** |  |  |  |
| Bones and Joints (23000)^b^ | 11 | 3.5% |  |
| Soft Tissue including heart (24000) | 51 | 4.3% |  |
| Eye and Orbit (30000) | 8 | 2.4% |  |
| Mesothelioma (36010) | 9 | 1.6% |  |
| Bone Marrow (37000) | 19 | 0.3% |  |
| Unknown primary (37000) | 68 | 4.7% |  |
| Percentage by disease site with IMD for patients diagnosed with primary cancer between 2010 and 2012. Follow-up for IMD ended on December 31, 2019, thereby allowing up to 7 years of followup for all patients. Disease sites where <6 IMD events occurred are not shown.  ^a^ recategorized the SEER recodes 20010-20100, 22010, 22020, or 32010 based on topography as follows:  Lip: C000-C003, C006-C009  Oral cavity: C003-C005, C050, C020-C024, C028-C029, C030-C049, C060-C069, C140-C149  Oropharynx: C010-C019, C051-C059, C090-C099, C100, C102-C109  Salivary glands: C079, C080, C081, C088, C089  Nasopharynx: C11 (same as recode)  Hypopharynx: C129, C130-C139 (same as recode)  Nose and nasal sinus: C300, C301, C310, C311, C313, C318, C319, C312  Larynx: C101, C320-C323, C328, C329  Thyroid: C739 (same as recode)  ^b^ brain metastasis only defined using C793 since primary treatment (e.g. radiation) may overlap with definition. For SEER recode 2300 if located within the bones of the skull or face and for SEER recode 32020 excluding adrenal gland, parathyroid gland, or thymus gland (predominately the pituitary and pineal glands).  ^c^ recategorized as adrenal gland (C749, C740-C741), thymus (C379), and parathyroid gland (<50 cases; C750) | | | |

# Figure S1: Classification of missing comorbidity information as absence of comorbidity based on overall survival pattern


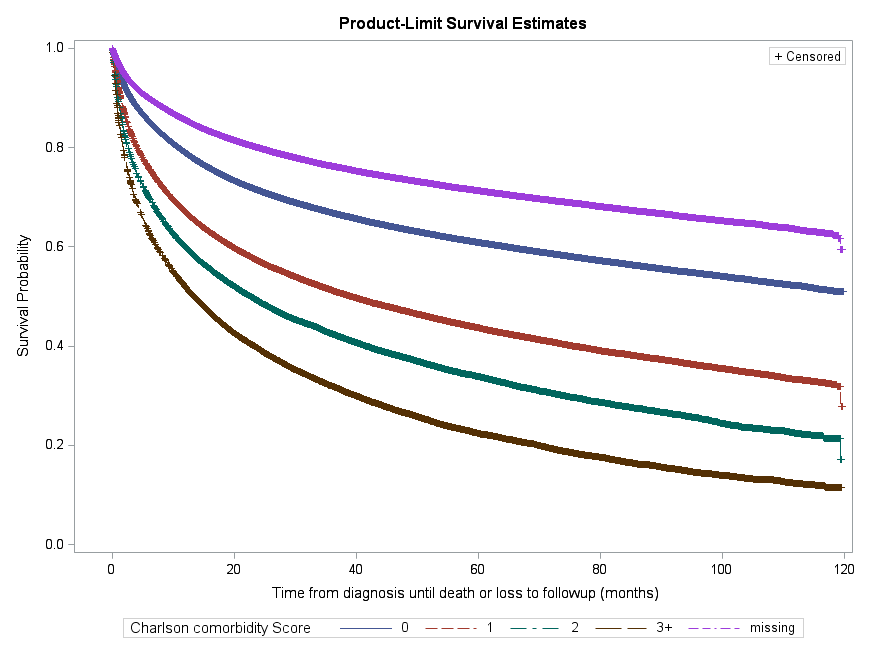


# Figure S2: Validation of metastasis definition using overall survival as an outcome indicator


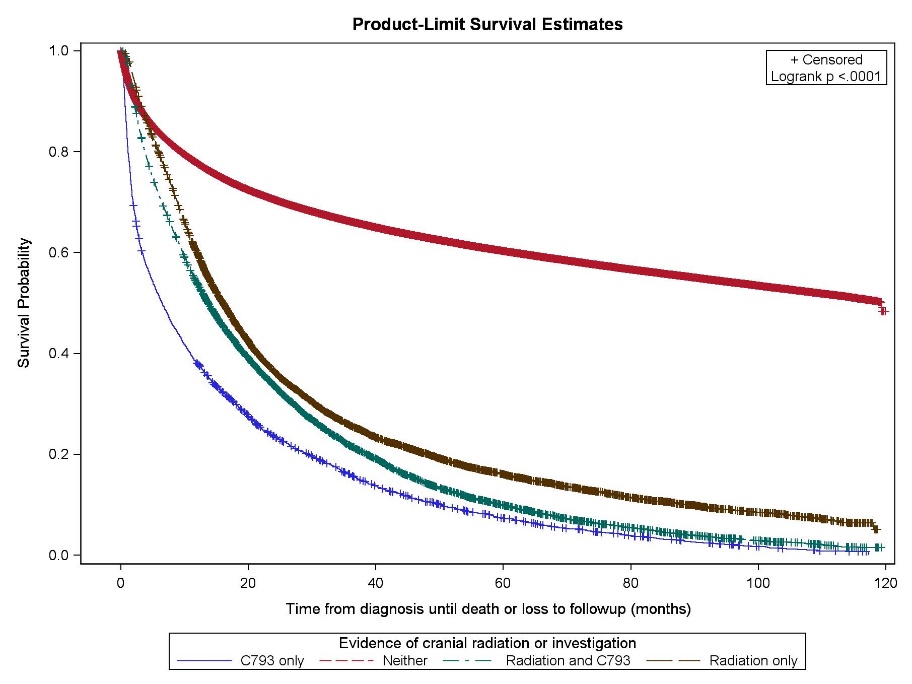


# Figure S3: Relationship between IMD and metastasis

In these figure we plot the proportion of patients developing IMD by the time until IMD is developed, by disease site. We further group primary disease groups by 1-year mortality. The purpose of these plots was to present the summary estimates from Table 2 visually and enable hypothesis-generation regarding the potential link between IMD and overall survival

A) all cancers


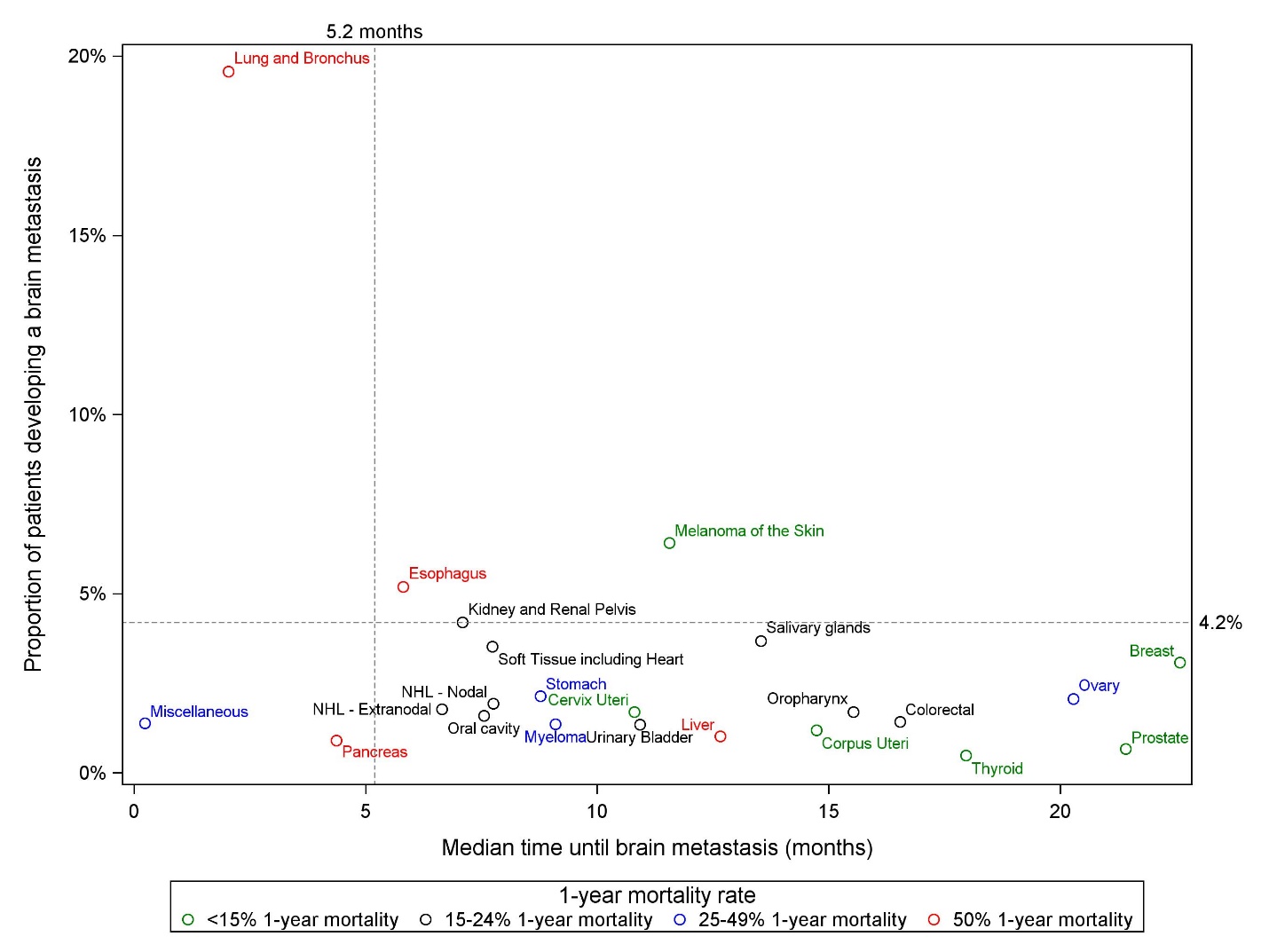


B) Lung cancer by stage


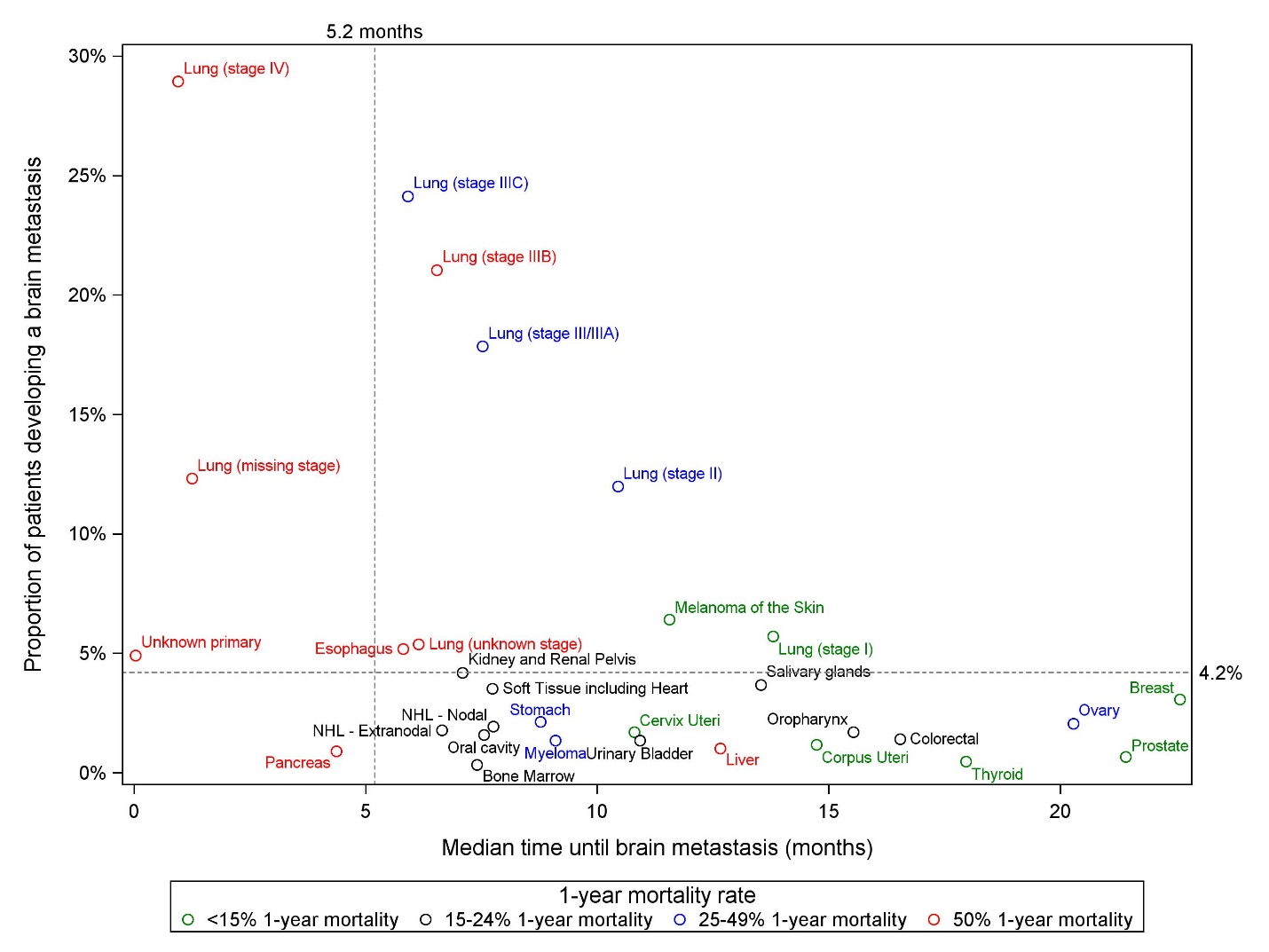


C) Breast cancer by stage


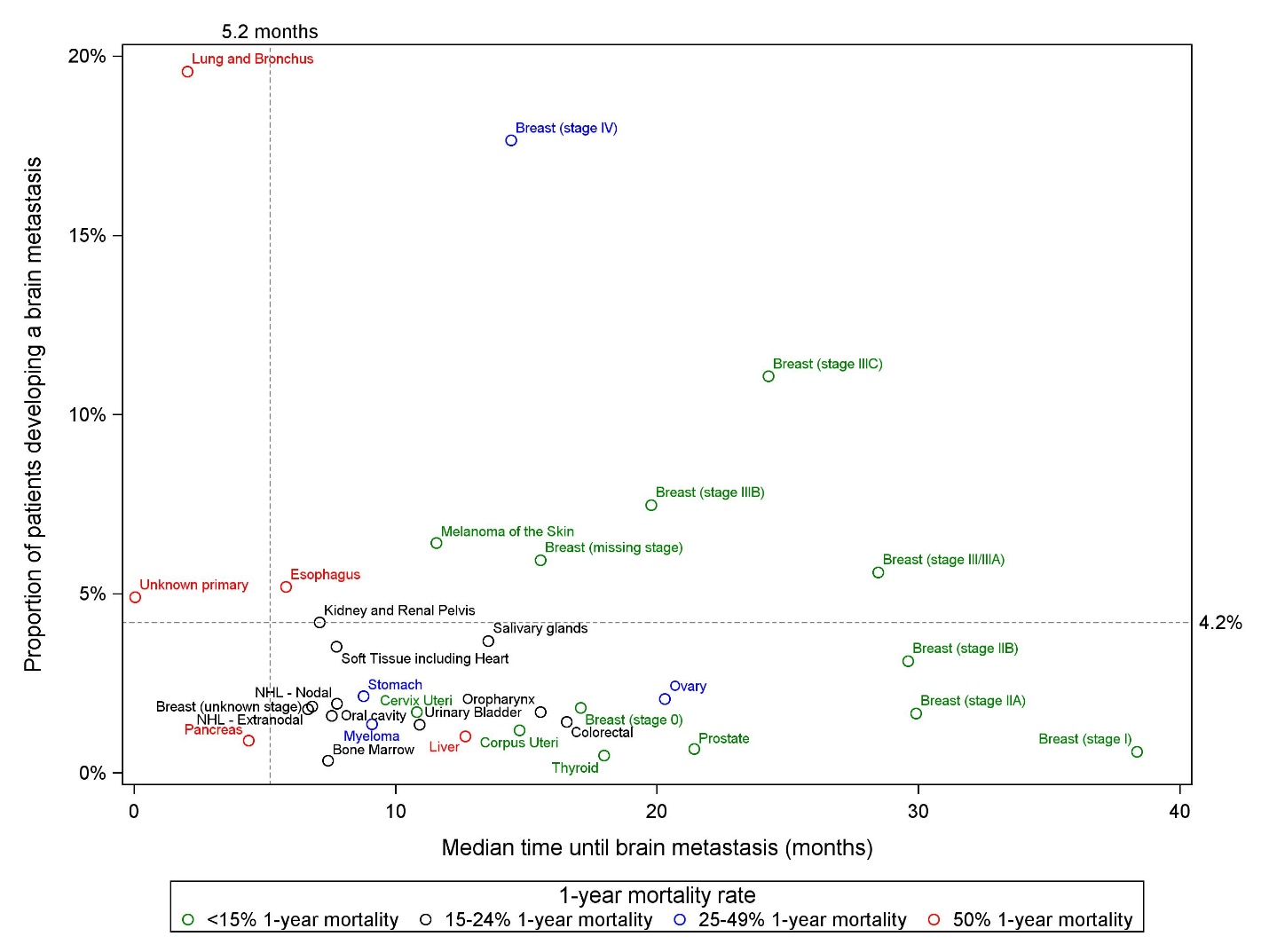


D) Prostate cancer by stage


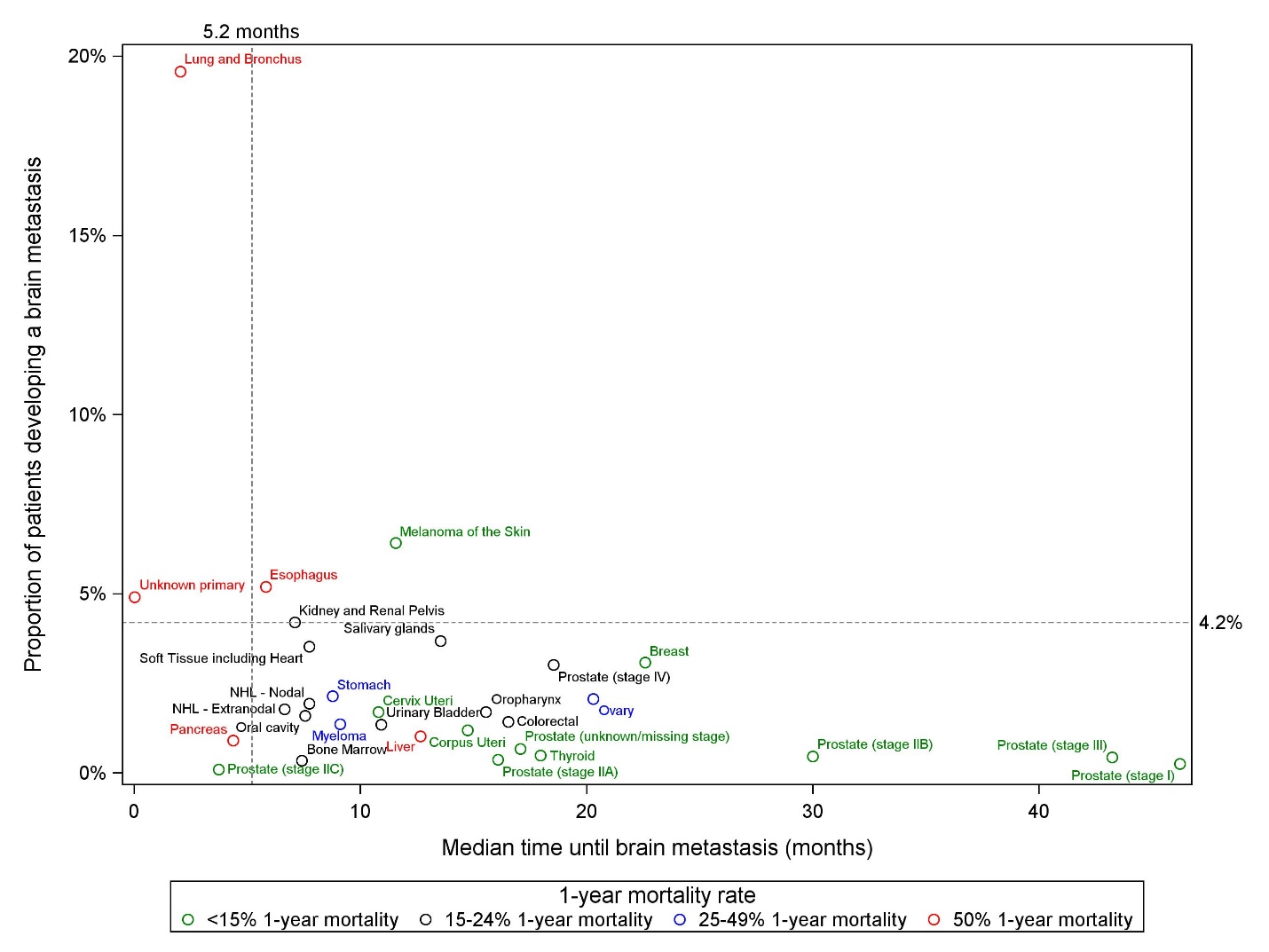


E) Colorectal cancer by stage


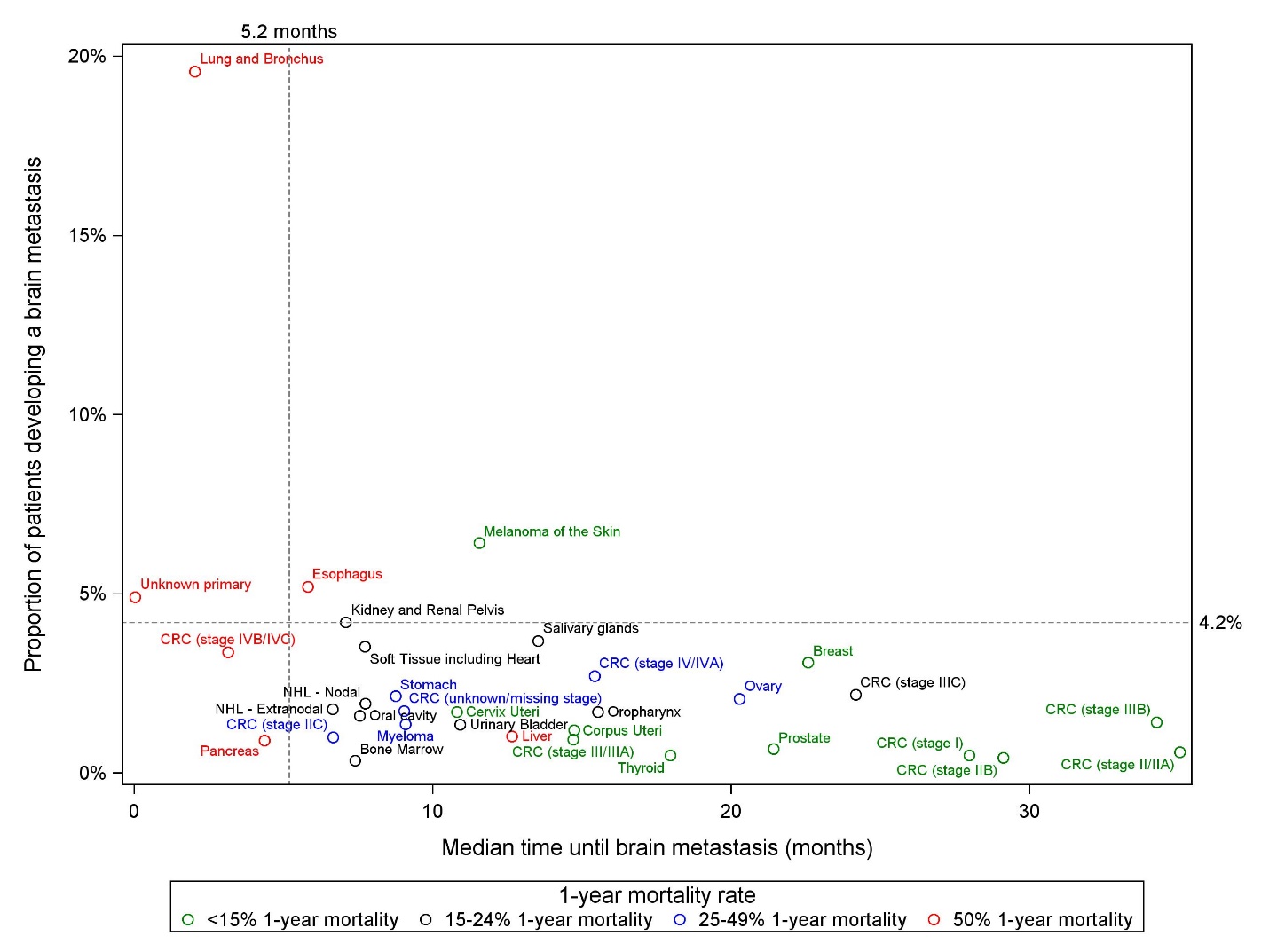


# Figure S4: Funnel plot for probability of IMD


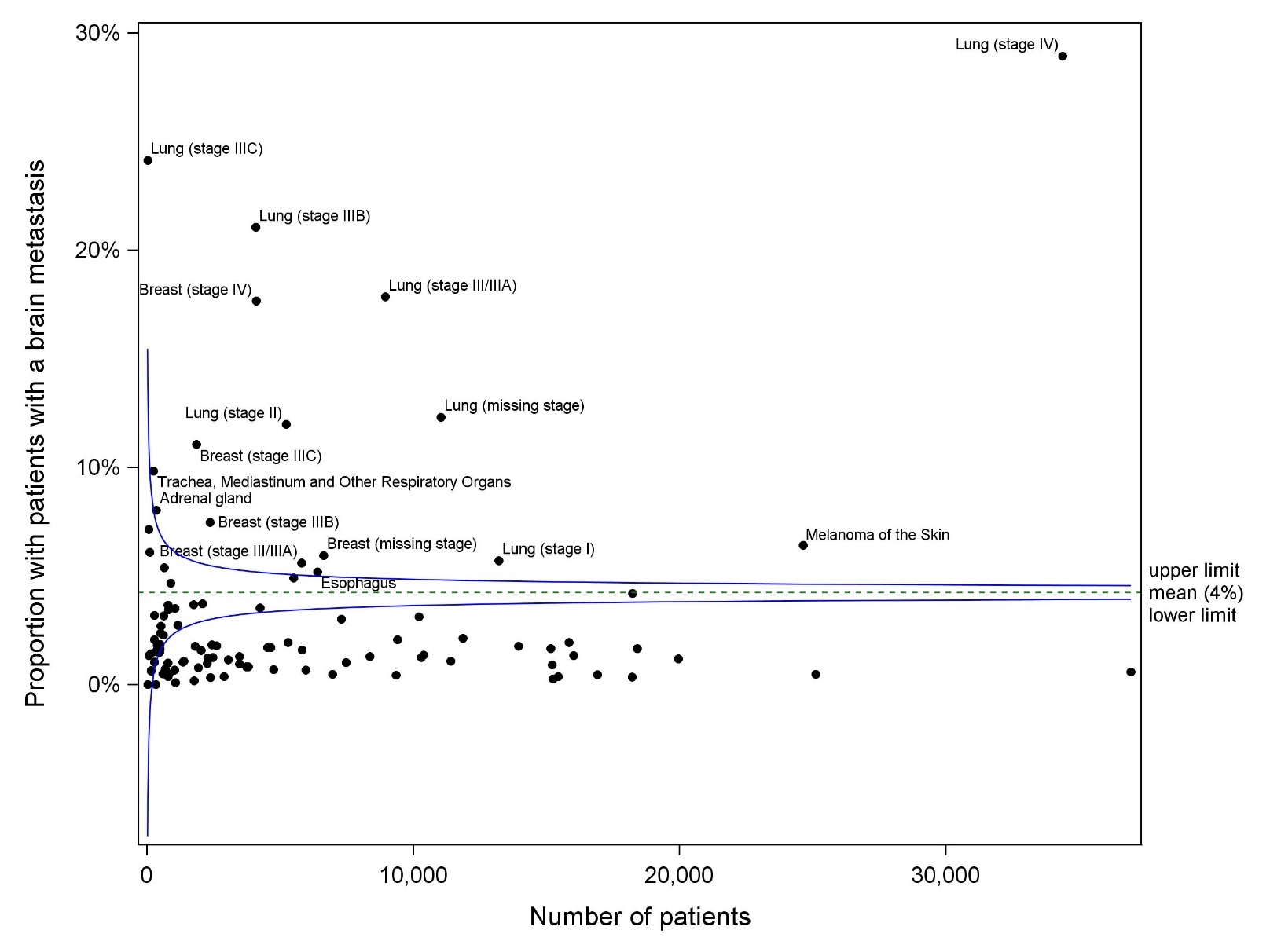


# Figure S5: Intracranial metastatic disease by cancers diagnosed 2010-2012

A)

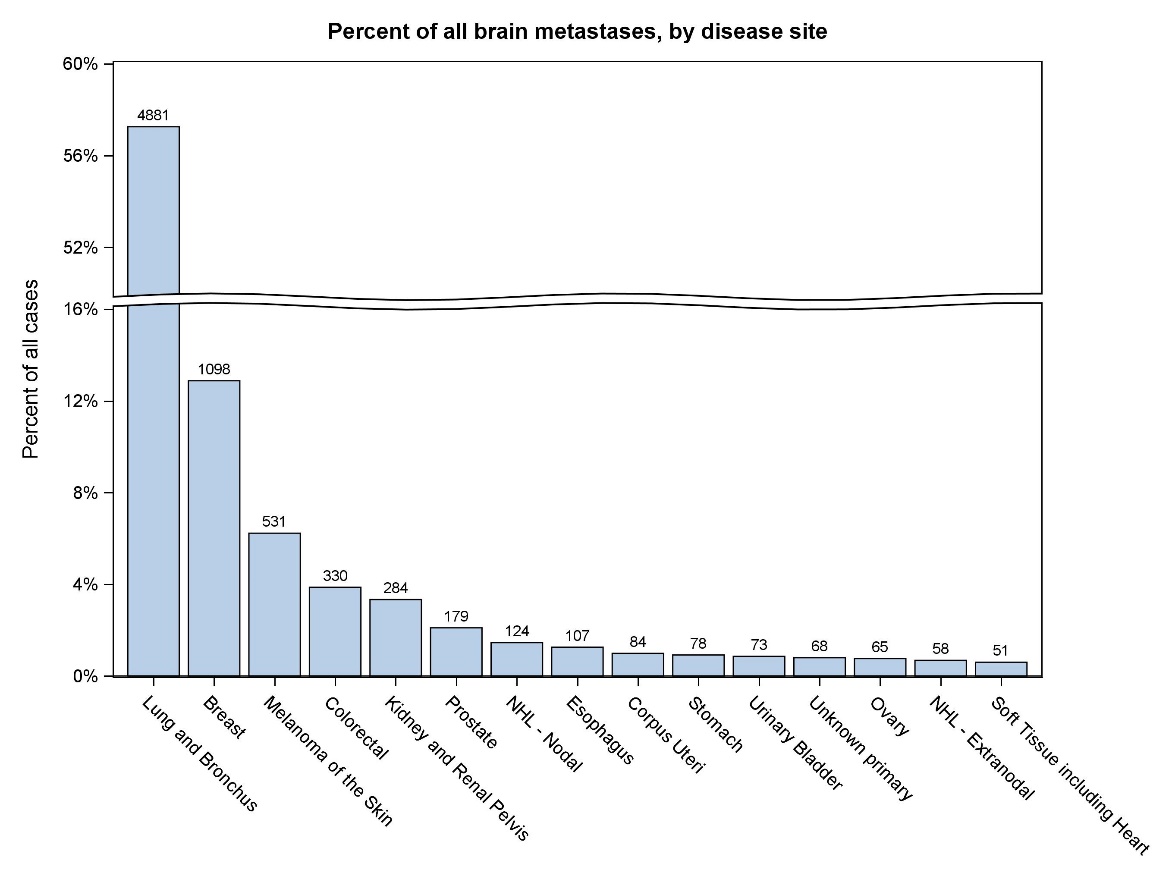


B)

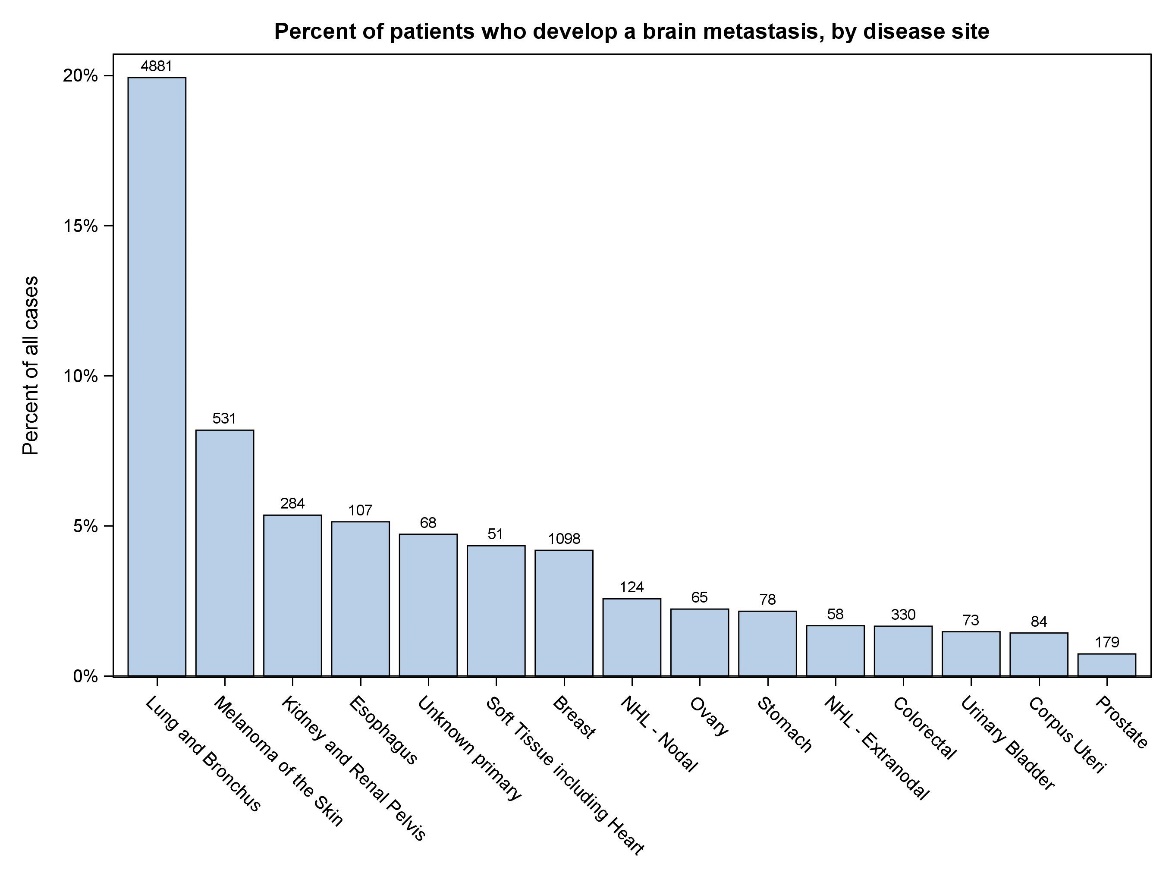

Supplement: vdaa178_suppl_Supplementary_Material [file vdaa178_suppl_supplementary_material.docx]
